# TA-BI: A Formal Observation-to-Decision Architecture for Trust-Aware Routing Support in Wireless and Mobile Wireless Sensor Networks

**DOI:** 10.3390/s26175422

**Published:** 2026-08-27

**Authors:** Wiesław Jabłoński, Ksawery Krenc, Adam Kawalec

**Affiliations:** 1 WB Electronics S.A., 129/133 Poznańska Street, 05-850 Ożarów Mazowiecki, Poland; 2 Institute of Missile Technology and Mechatronics, Faculty of Mechatronics, Armament and Aerospace, Military University of Technology, 2 gen. Sylwestra Kaliskiego Street, 00-908 Warsaw, Poland; ksawery.krenc@wat.edu.pl; 3 Faculty of Mechatronics, Armament and Aerospace, Military University of Technology, 2 gen. Sylwestra Kaliskiego Street, 00-908 Warsaw, Poland; adam.kawalec@wat.edu.pl

**Keywords:** wireless sensor networks, mobile wireless sensor networks, sensor network security, reactive routing, trust-aware routing, formal architecture, observation-to-decision contract, routing decision support, evidence provenance

## Abstract

TA-BI (trust-aware behavioral intelligence) is a conceptual–formal observation-to-decision contract for trust-aware routing support in reactive multi-hop wireless sensor networks (WSNs) and mobile wireless sensor networks (MWSNs). It specifies evidence provenance and availability, separates current Behavioral Information from historical node Trust, maps these states to route candidates, and controls when RouteScore may affect a native routing decision. In the reference AODV adapter, declared validity checks, destination-sequence freshness, and hop-count precedence are applied before RouteScore on a finite frozen candidate set. A complete common component basis prevents asymmetric missing evidence from favoring a candidate; a closed gate disables TA-BI re-ranking but retains a total routing outcome through baseline ordering or native fallback. Nine propositions establish bounded contract properties, including candidate-admissibility preservation rather than equivalence of the complete AODV transition system. A deterministic Python oracle evaluates ten groups, with explicit T3/T4 subcases for stale, version-inconsistent, missing, low-confidence, and restored evidence. The evidence is separated into formal correctness, executable conformance, historical integration reachability, and empirical network performance. The supplied formal and deterministic artifacts support only the first two levels; the bounded historical OMNeT++/INET record supports integration reachability only. Packet delivery, delay, throughput, energy, overhead, scalability, and mobility performance are not evaluated.

## 1. Introduction

Wireless sensor networks (WSNs) organize sensing, processing, and relaying nodes around the delivery of measurements under energy, memory, processing, radio-range, and link-quality constraints. Mobile wireless sensor networks (MWSNs) add the mobility of sensing nodes, relays, sinks, or mixed roles. Mobility can improve coverage, but it also changes neighborhoods, route validity, evidence age, and forwarding observability [1,2].

MWSNs share decentralized multi-hop communication and topology dynamics with mobile ad hoc networks (MANETs), but the two domains are not treated as synonymous. The scope is sensor-oriented: constrained nodes generate, relay, or collect measurements toward a sink, gateway, or data-collection endpoint. MANET research supplies protocol and trust-management lineage; the application claims remain limited to WSNs and MWSNs.

Trust-aware routing must connect current observations and historical reliability to candidate selection without treating unavailable evidence as an ordinary neutral value. Packet loss, delay, link change, and forwarding behavior have different assessed objects and time horizons; their provenance and availability therefore remain explicit.

The principal contribution of TA-BI is an observation-to-decision contract, not a new scalar trust metric and not a replacement routing protocol. The contract specifies which evidence may be consumed, which object each state assesses, when missing or stale evidence becomes unavailable, how candidate scores are compared on a common basis, and how the reference adapter applies declared AODV admission, precedence, revalidation, and fallback rules.

Behavioral Intelligence denotes the overall capability set of TA-BI, whereas Behavioral Information (BI) is one current node-level metric. BI and NeighborBI use the current evidence horizon, while Trust retains bounded historical memory per assessed node. RouteBI and RouteScore belong to candidate (i,d) in the AODV adapter or route *r* in a complete-path adapter. Candidate identity and route-instance version are separate objects.

AODV is the reference adapter because its RREQ/RREP exchange exposes explicit route candidates [3,4]. The adapter applies destination-sequence freshness first and hop count second. RouteScore is consulted only inside the resulting native tie class and only when that class has a complete common component basis. Otherwise, the declared deterministic baseline tie policy is used. Complete-path adapters apply an analogous full-coverage rule to weakest-link Trust.

Four evidence levels delimit the inferences. Formal correctness asks whether a proposition follows from declared assumptions and definitions. Executable conformance asks whether deterministic code exercises the specified state and decision rules. Integration reachability asks only whether modules and route-lifecycle events were observed to connect in a bounded implementation trace. Empirical network performance requires repeated controlled runs and uncertainty reporting. Evidence at one level does not establish a claim at a higher level.

**Scope of application.** The target class comprises multi-hop WSNs and MWSNs in which sensing, relay, or gateway nodes participate in reactive route discovery toward a sink, gateway, or data-collection endpoint. The AODV specialization is used as a reference adapter because it exposes explicit next-hop–destination candidates and native freshness and hop-count rules. MANET research supplies routing and trust-management mechanisms, but general-purpose MANET deployment is outside the evaluated application scope.

### 1.1. Research Question and Contributions

**Research question (RQ).** Can current and historical evidence in reactive multi-hop WSNs and MWSNs be transformed into reproducible routing-candidate assessments while retaining provenance, state-level meaning, common-basis treatment of missing evidence, and respecting protocol-specific candidate-admissibility conditions under sensor-node resource constraints and possible mobility?

The study addresses the RQ through four analytical objectives:

**AO1:**  Define assessed objects, evidence classes, state domains, availability, confidence, completeness, versions, and update order.
**AO2:**  Define node-to-candidate mappings and an adapter that returns either a revalidated selection or native fallback.
**AO3:**  Prove boundedness, sensitivity, common-basis neutrality, ordering, admissibility, totality, convergence, and local switching properties.
**AO4:**  Link the formal contract to deterministic tests and a bounded integration trace without extending the inference to performance superiority.

The principal contributions are as follows:

1. A typed state model that distinguishes evaluator *o*, node *i*, destination *d*, candidate *c*, complete route *r*, and route-instance version $\nu_{i,d}$.
2. A canonical missing-evidence rule and an AODV common-mask rule that prevents candidates with different evidence bases from being compared by RouteScore.
3. A complete-path Trust-completeness rule that prevents missing weak nodes from increasing decision-usable route Trust.
4. An AODV adapter in which RouteScore cannot override fresher sequence information or a lower hop count.
5. Deterministic event ordering, versioned snapshots, final revalidation, and a total Select/Fallback result.
6. Nine propositions, incremental computation, storage, and communication accounting, a ten-group oracle with explicit degraded-evidence subcases, and a five-profile decision-semantic AODV–TA-BI comparison.
7. A four-level evidence taxonomy and a documented historical model-to-module trace whose inference is limited to integration reachability.
8. An explicit application boundary that treats reactive multi-hop WSNs and MWSNs as the target class and retains MANET mechanisms only as theoretical and protocol lineage.

### 1.2. Scope and Inferential Boundary

TA-BI supports assessment after the native routing protocol has established candidate feasibility. The application scope is limited to reactive, multi-hop WSNs and MWSNs in which constrained sensing, relay, or gateway nodes may expose more than one feasible next-hop candidate toward a sink, gateway, or data-collection endpoint. A deployment may be static or may include mobile sensors, mobile relays, a mobile sink, or mixed mobility.

Route discovery, repair, authentication, identity management, integrity protection, loop prevention, medium-access control, duty cycling, and energy management remain external responsibilities. The results establish bounded formal properties, executable branch-level conformance, and historical integration reachability at their respective evidence levels. They do not establish superior delivery, delay, energy, recovery, scalability, mobility tolerance, or attack-detection performance. Those outcomes require paired repeated WSN/MWSN experiments with identical scenarios and seeds.

The article does not claim a completed adapter for LEACH cluster-head election, RPL DODAG construction, CTP collection-tree maintenance, strictly sink-only forwarding, or duty-cycle-aware MAC coordination. Residual energy is not a direct RouteScore component in the reference profile. It may enter a future profile only through an explicitly defined, normalized, and validated component whose use preserves the native protocol contract.

The terms *lightweight*, *scalable*, *low overhead*, and *real-time* are therefore design targets, not empirical conclusions. The reference adapter is specified for AODV in the stated sensor-network class. General-purpose MANET deployment and every other routing family require their own assessed object, admissibility rules, decision point, timing contract, resource accounting, and conformance record.

### 1.3. Formal Assumptions

The results use the following assumptions:

**A1:**  Every defined primitive communication-quality or reliability input belongs to [0,1]; identifiers, counters, time quantities, and protocol fields use the declared domains.
**A2:**  Aggregation weights are non-negative. A composite state is defined only when its active-weight sum is positive; normalized active weights sum to one.
**A3:**  Every consumed component carries availability, age, confidence, provenance, and version metadata. Missing, stale, unresolved, unsupported, or version-mismatched evidence is inactive.
**A4:**  Each frozen AODV comparison set lies within a modular sequence-number interval of width less than $2^{31}$, or the native implementation supplies resolved freshness classes.
**A5:**  The native protocol establishes feasibility before TA-BI ordering and revalidates it immediately before route installation or switching.
**A6:**  Historical update coefficients satisfy $0<\delta_{X,\min}\leq \delta_{X,i,k}\leq \delta_{X,\max}\leq 1$.
**A7:**  The no-immediate-reversal result assumes score variation $\rho <\varepsilon_{\mathrm{sw}}$ between consecutive decision epochs.
**A8:**  Each local execution context serializes equal-time events by the declared priority and local serial number, closes an observation window atomically, and publishes candidate state as one versioned snapshot.
**A9:**  The canonical AODV adapter opens its common tie-set gate only for a complete shared component mask. A canonical complete-path adapter exposes weakest-link candidate Trust only with full required-node coverage.
**A10:**  The target deployment is a multi-hop WSN or MWSN with bounded energy, memory, processing, and radio resources. Mobility may be absent or may affect sensing, relay, sink, or gateway nodes. This domain assumption bounds the application claims and resource accounting; it is not required for the algebraic range proofs under A1–A3.

## 2. Related Work

The review is organized around the narrowed application scope. It first considers trust management in WSNs, then trust-aware routing and mobility-aware routing in WSNs and MWSNs. MANET mechanisms are retained only where they provide the behavioral-monitoring, reputation, AODV, or verification lineage needed by the reference adapter. Reported performance values are not compared across papers because their topologies, radios, workloads, mobility models, attacks, and simulators are not equivalent.

### 2.1. Review Method and Selection Boundary

The selective narrative review was updated for this revision in July 2026. The corpus was organized in three passes. The first pass identified trust, reputation, anomaly-monitoring, and resource-aware routing mechanisms in WSNs. The second selected WSN, MWSN, underwater-sensor, and sensor-oriented IoT studies that expose a trust-to-route or mobility-to-route rule. The third retained MANET/AODV mechanisms and formal protocol analyses only when they were necessary to define behavioral lineage, destination-sequence freshness, admissibility, or loop-freedom obligations.

The search expressions combined *wireless sensor network*, *mobile wireless sensor network*, *mobile sensor routing*, *trust-aware routing*, *sensor network security*, *behavioral monitoring*, *AODV trust*, *reactive routing*, *candidate ranking*, *missing evidence*, *routing invariant*, and *loop freedom*. A source was retained when it contributed an evidence model, trust update, routing-candidate rule, mobility treatment, protocol-state interpretation, implementation result, or formal correctness argument. Primary sources and protocol specifications were preferred.

The comparison matrices use a documented coding rule. **Y** denotes an explicit element connected operationally to the routing contract. **P** denotes partial, inferred, scheme-specific, or incomplete treatment. **N** means that no explicit treatment was found in the reviewed source. **N/A** marks an item outside the source’s scope. The coding was repeated in a second source-by-source verification pass. Ambiguous cells were downgraded to P. Supplementary File S4 records the primary network domain, operational definition, source locator, code, and verification note for every matrix cell. The review is not systematic; absence claims are restricted to the reviewed corpus and the coded contract dimensions.

### 2.2. Trust Management in Wireless Sensor Networks

TARF and LDTS demonstrate that trust can guide forwarding and clustering decisions under WSN resource constraints [5,6]. Their contributions include local observation, reputation exchange, and routing policies adapted to sensor-network operation. They motivate the separation of current measurements, accumulated reliability, communication cost, and route use, but their internal state objects and absence rules remain specific to each scheme.

Anomaly and intrusion-detection research provides additional evidence sources for WSN security [7,8,9,10,11,12,13,14,15]. Such outputs do not constitute a route-selection contract by themselves. Their use requires an explicit assessed object, observation horizon, provenance rule, availability state, confidence interpretation, and protocol decision point.

### 2.3. Trust-Aware Routing in WSNs and MWSNs

Recent sensor-network routing broadens the evidence and optimization space. A void-avoiding underwater WSN scheme combines direct, indirect, and environmental trust with two-hop availability checks [16]. GTR combines GAN-based trust evaluation with Q-learning route selection [17]. ACTAR integrates clustering, multi-objective cluster-head selection, and trust-aware next-hop choice [18]. A distributed fuzzy protocol incorporates trust into multipath WSN routing [19], while TEAHR combines hierarchical routing, trust, and energy considerations [20]. Context-aware trust prediction illustrates how contextual evidence may guide intermediary selection in opportunistic IoT systems [21].

MWSN routing introduces an additional state-change source: mobility of sensors, relays, sinks, or mixed roles. Surveys and implemented routing studies emphasize topology repair, collection continuity, resource constraints, and the distinction between mobile-node and mobile-sink cases [1,2]. These requirements strengthen the need for evidence aging, route-instance versioning, and final revalidation. One AODV profile does not represent every MWSN routing family; the defined profile is limited to reactive multi-hop operation in which route discovery exposes comparable candidates.

### 2.4. MANET Mechanisms Relevant to the AODV Adapter

Watchdog/Pathrater connected overheard forwarding to route avoidance, while CORE and CONFIDANT developed collaborative reputation in MANETs [22,23,24]. TAODV, AOTDV, T2AR, and direct-trust AODV variants illustrate protocol-specific ways of injecting trust into on-demand route selection [25,26,27,28]. These mechanisms are retained because they inform the AODV adapter and the interpretation of forwarding behavior. They are not presented as validation of TA-BI for the full MANET domain.

This distinction matters because a general-purpose MANET may support peer-to-peer traffic, heterogeneous mobile devices, and application goals unrelated to sensing. The target WSN/MWSN class instead retains sensor-oriented data acquisition, constrained resources, and sink, gateway, or collection-endpoint roles. MANET-derived ideas therefore enter as reusable mechanisms, not as an expansion of the application claim.

### 2.5. Protocol Verification and Adapter Motivation

Formal analyses show why an explicit adapter boundary is necessary. HOL/SPIN analysis exposed ambiguities in distance-vector standards [29]. AWN and Isabelle/HOL established AODV route-correctness and loop-freedom obligations for defined interpretations [30,31]. TA-BI does not replace those obligations. It constrains its score to a native-equivalent tie class and delegates final feasibility to the protocol.

### 2.6. Comparative Architecture Matrix

Table 1 and Table 2 summarize the representative sources. The added network-domain column prevents WSN, MWSN, underwater-sensor, opportunistic-IoT, and MANET evidence from being interpreted as one homogeneous validation corpus. The matrices are evidence maps, not league tables. A Y for TA-BI indicates an explicit element in the proposed contract; it does not indicate superior network performance.

### 2.7. Research Gap

The WSN and MWSN literature provides strong component mechanisms for observation, reputation, mobility response, learned trust, clustering, multipath routing, and resource-aware forwarding. The MANET and AODV literature adds behavioral-monitoring lineage and protocol-specific correctness obligations. The remaining gap lies in the interface between these elements. No single reviewed approach explicitly combines all of the following for the target reactive sensor-network class:

(i) Typed evidence semantics with provenance, age, confidence, version, and absence handling.
(ii) A level-consistent mapping from current node evidence and historical node Trust to a route candidate.
(iii) Protocol-constrained candidate selection with native precedence, common-basis comparison, final revalidation, and total fallback.
(iv) Analytical claims linked to executable boundary tests, implementation responsibilities, and resource-cost accounting.

TA-BI contributes this combined contract. Its novelty is architectural and compositional. It specifies how an estimator may be replaced without changing the assessed object, missing-evidence semantics, sensor-network application boundary, or authority of the routing protocol.

**Figure 1:**
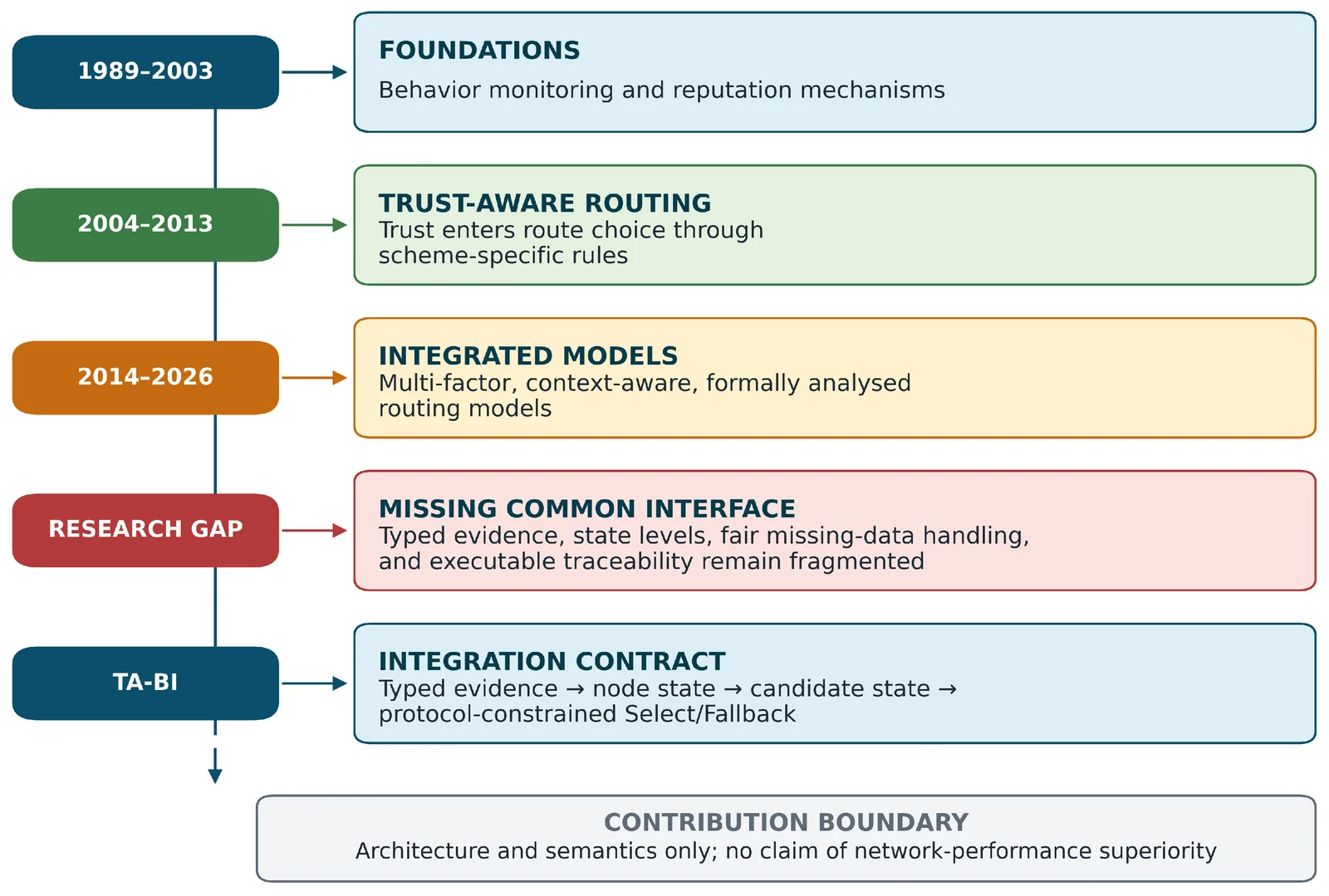
Evolution from component mechanisms to the TA-BI integration contract. The diagram identifies the missing common interface and bounds the contribution to architecture and compositional semantics; it does not imply network-performance superiority.

Figure 1 summarizes the progression from component mechanisms to the TA-BI integration contract. MANET-derived mechanisms are shown only as theoretical and protocol lineage; the application claims remain limited to WSNs and MWSNs.

The diagram summarizes the progression from behavioral monitoring and trust-aware routing to a combined observation-to-decision contract. MANET-derived mechanisms are retained as historical and protocol lineage, whereas the evaluated application scope is limited to WSNs and MWSNs. The figure does not assert quantitative superiority or priority for individual component mechanisms.

## 3. Materials and Methods: TA-BI Architecture and Design Principles

### 3.1. Design Objectives

TA-BI follows five design objectives. First, the declared native AODV admission and commitment checks are applied before TA-BI ranking. Second, local observation is primary and indirect evidence is optional, aged, and provenance-controlled. Third, each state has an explicit object, horizon, availability rule, and point of use. Fourth, missing or failed analytical state produces a baseline decision rather than a fabricated score. Fifth, every adapter must expose its incremental processing, storage, timing, and communication cost. These objectives define a conformance target; their operational efficiency remains an empirical property.

### 3.2. Overall TA-BI Architecture

TA-BI places the analytical process between local behavioral observation and the native candidate-selection point of the underlying routing protocol. The common analytical core computes interpretable communication-quality and operational-reliability states, whereas the protocol-specific adapter exposes the resulting RouteScore to the routing protocol without assuming responsibility for route discovery, route maintenance, or route repair.

Five principal components form a single chain of responsibility. The Observation Layer acquires and classifies observable communication events. The Behavioral Information Layer computes BI, NeighborBI, and RouteBI. The Trust Assessment Layer estimates the temporally aggregated Trust of individual nodes. The Route Decision Engine combines the available route-level and node-level assessments into RouteScore. Finally, the Protocol-Specific Integration Adapter verifies candidate admissibility and controls whether and how RouteScore is applied within the native candidate-selection procedure.

**Figure 2:**
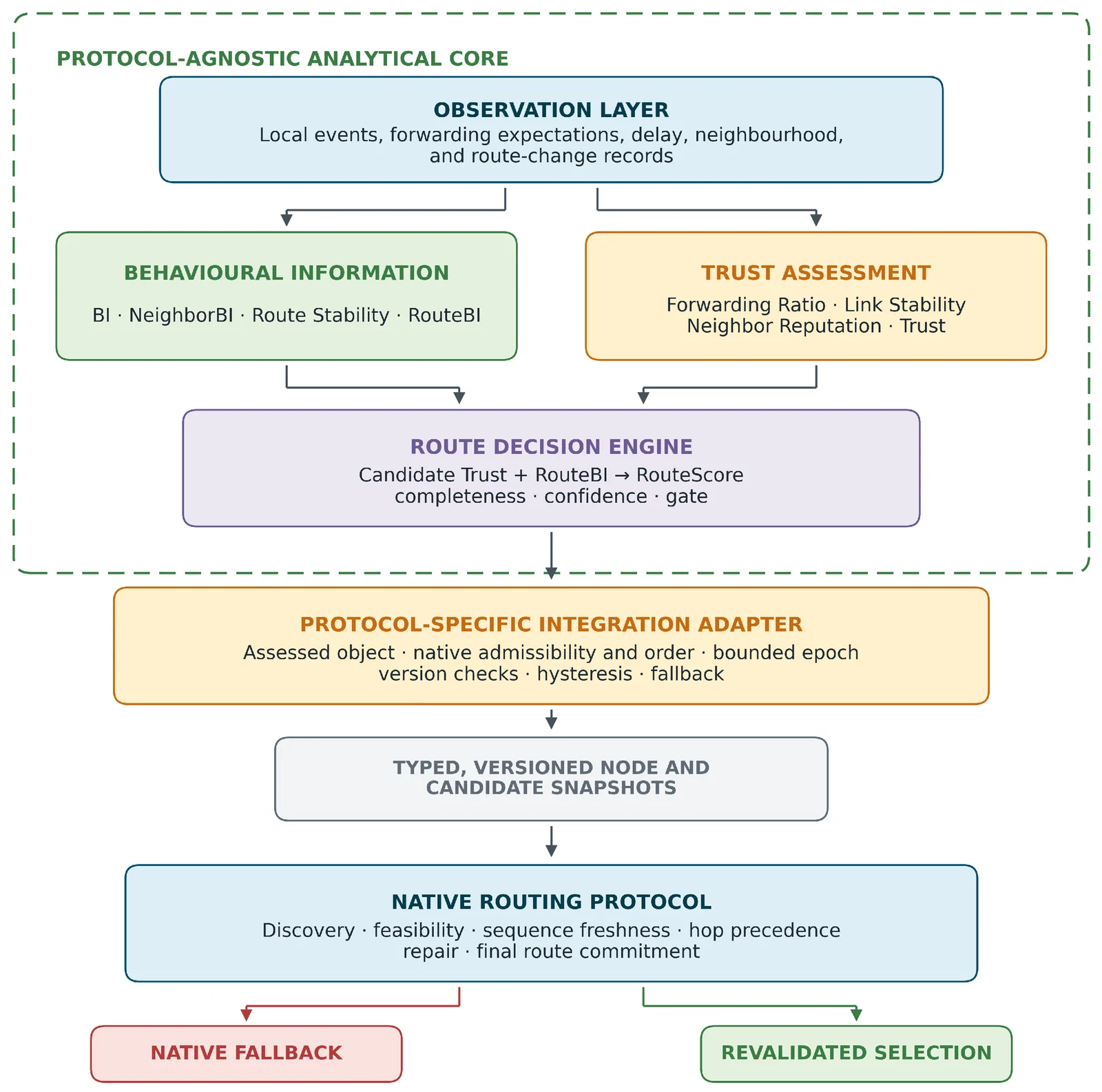
TA-BI architecture and authority boundary among the protocol-agnostic analytical core, protocol-specific adapter, and native routing protocol. Solid arrows denote permitted information flow; the dashed enclosure marks the analytical core.

The dependencies and information flow among these components are illustrated in Figure 2.

The separation of observation, behavioral estimation, trust assessment, score computation, and decision execution enables the individual modules, instrumentation mechanisms, evaluation metrics, and protocol-integration policy to be examined independently. Consequently, each change in RouteScore can be traced to a specific event class, observation source, update rule, and analytical component.

The protocol-specific adapter encapsulates all coupling between the common TA-BI analytical core and the underlying routing protocol. It defines the assessed routing object, the protocol state available to the analytical layer, the candidate-selection integration point, the declared native candidate-validity and ordering checks applied before RouteScore, and the cost of any required protocol extensions. Changes to protocol interfaces, maintained state, control messages, or memory requirements must therefore be explicitly documented and evaluated for each implementation.

### 3.3. Functional Components of the TA-BI Framework

This section defines the responsibility of each component; their execution order is specified in the operational-flow subsection below. The Observation Layer records transmissions, receptions, expected forwarding, delays, neighborhood state, and canonical changes of the active route. Passive observation is the primary source; indirect data are optional and retain provenance.

The Behavioral Information Layer normalizes local packet loss and delay at the node level, aggregates NeighborBI, and computes RouteBI while incorporating Route Stability. The Trust Assessment Layer updates the Forwarding Ratio, Link Stability, Neighbor Reputation, and Trust using distinct memory horizons.

The Route Decision Engine combines RouteBI and Trust into RouteScore. The adapter applies confidence gating and declared native candidate-validity and ordering checks before using the score as a secondary criterion or tie-breaker.

### 3.4. TA-BI Operational Flow

After a local observation window closes, a node sequentially classifies events, updates BI and admissible indirect information, estimates Trust, computes RouteBI and RouteScore, and passes the result to the adapter.

The adapter revalidates candidate feasibility, evaluates confidence, applies hysteresis, and submits the decision to the protocol’s native mechanism. The cycle is local, asynchronous, and independent of a global clock.

**Figure 3:**
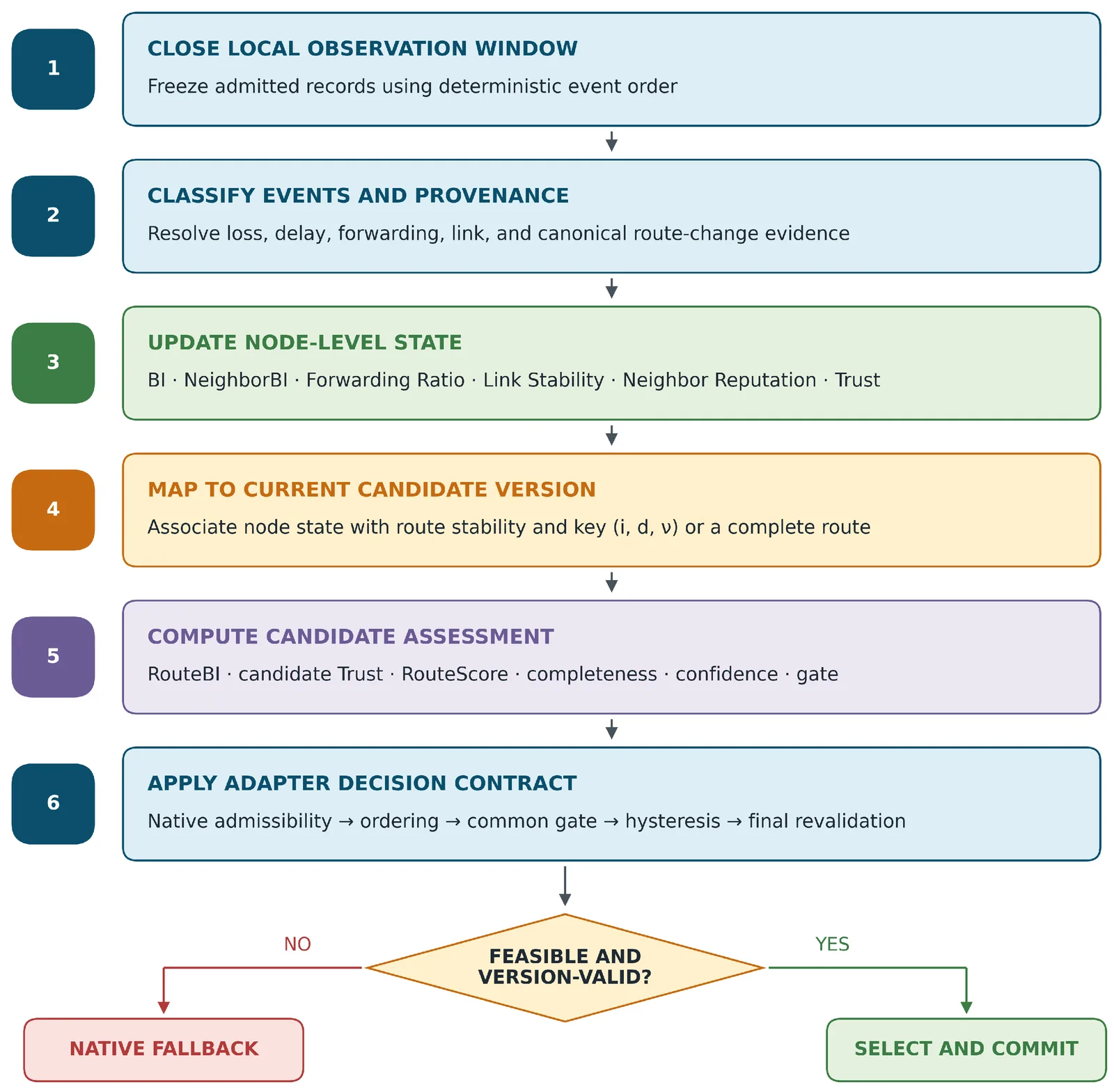
Deterministic observation-to-decision flow. Numbered blocks show the processing order; the terminal branch returns either a revalidated selection or native fallback.

Figure 3 presents this end-to-end execution order and distinguishes revalidated route selection from controlled native fallback.

The confidence-gating states, data-aging policy, and controlled fallback are defined in detail in Section 5.4.

## 4. Formal Metric Architecture

### 4.1. Overview of the Assessment Model

TA-BI distinguishes between the node level, the local routing-candidate level, and the generic adapter interface. This separation preserves the node-level semantics of Trust while assigning RouteBI and RouteScore to a specific route toward a particular destination.

All primary TA-BI states are local to a fixed evaluator *o*. Compact notation suppresses superscript (o). When evaluator identity matters, Equation (1) gives the full forms:

(1) $${\mathrm{BI}}_{i}^{\left(o\right)}\left(t\right),\;{\mathrm{NeighborBI}}_{i}^{\left(o\right)}\left(t\right),\;{\mathrm{Trust}}_{i}^{\left(o\right)}\left(t\right).$$

Every compact state therefore denotes an evaluator-local assessment.

In the formal model, *o* denotes the fixed local evaluator, *j* an observer or reporter supplying direct or indirect evidence, *i* the assessed node, *d* the routing destination, *c* a generic candidate admitted by the adapter, *r* a complete route, and *t* time. In the reference AODV adapter, node *i* is the next hop, whereas the pair (i,d) identifies the current local candidate toward destination *d*.

**Definition 1**  (Current evidence horizon)**.** *For component X concerning assessed object z, the current evidence horizon at time t is defined by Equation (2):*

(2) $$\begin{array}{cc} H_{z}^{X}\left(t\right)=\{e:\; & e\;\mathrm{concerns}\;\left(X,z\right),\;t_{e}\in W_{X}\left(t\right),\\ \; & e\;\mathrm{passes}\;\mathrm{age},\;\mathrm{resolution},\;\mathrm{provenance},\\ \; & \mathrm{confidence},\;\mathrm{and}\;\mathrm{version}\;\mathrm{checks}\}. \end{array}$$

*A value is* current *only when it is computed from records in $H_{z}^{X}\left(t\right)$. The most recently stored numerical value is therefore not automatically current; it may be retained for continuity while its availability indicator is zero.*

Table 3 distinguishes the object types, identifiers, and route-instance key used by the analytical core and the reference AODV adapter.

Candidate identity and route-instance version are distinct. The pair (i,d) identifies the local AODV candidate, whereas $\nu_{i,d}$ distinguishes successive installations or activations of that candidate association.

The conceptual metric architecture comprises the following:

- **RouteScore**—the final score exposed through the generic adapter interface; in AODV, it is indexed by the next-hop–destination pair (i,d);
- **RouteBI**—the behavioral assessment of a candidate; in AODV, it is indexed by (i,d), whereas full-path protocols use a route identifier *r*;
- **BI**—the local communication-quality assessment associated with assessed node *i*;
- **NeighborBI**—aggregated Behavioral Information concerning node *i* and originating from reporters *j*;
- **Trust**—the local, temporally updated assessment of the operational reliability of node *i*;
- **Neighbor Reputation**—the aggregated indirect reputation used to estimate the Trust of node *i*.

Trust is stored once per node. RouteBI and RouteScore retain destination-dependent next-hop and reconfiguration context under key (i,d).

**Figure 4:**
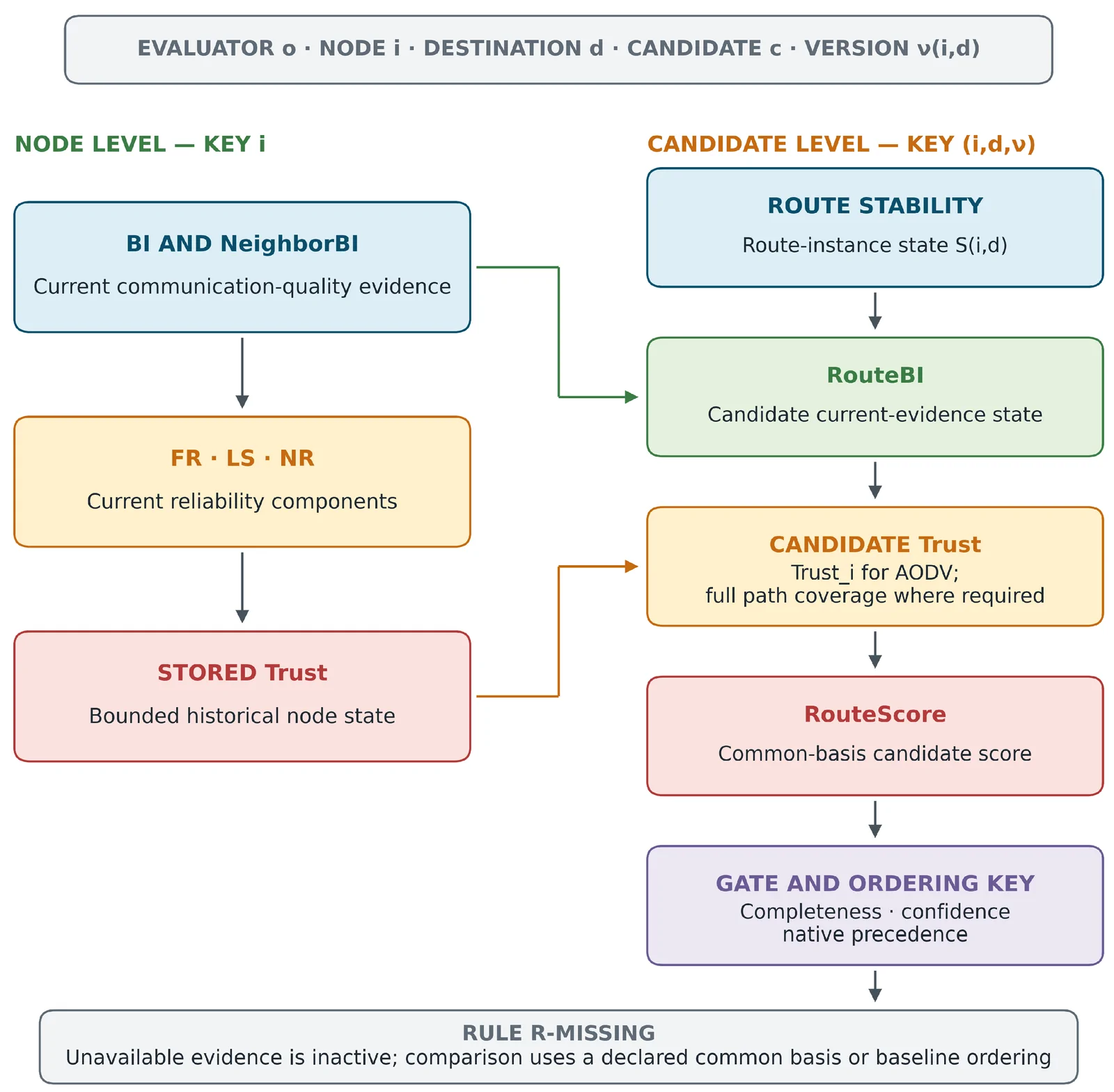
Separation of node-level state, candidate-level state, and adapter metadata. Solid arrows denote permitted information flow; they do not imply network-performance causality.

Figure 4 summarizes the separation between node-level state, candidate-level state, and adapter metadata.

Node-level BI, NeighborBI, Forwarding Ratio, Link Stability, Neighbor Reputation, and Trust are separated from candidate-level Route Stability, RouteBI, RouteScore, confidence, and gate metadata.

The generic symbol *c* remains part of the adapter contract. For the reference AODV specialization, the following mapping applies:

(3) $$\begin{array}{cccc} c & =c_{\left(i,d\right)}, & & \\ {\mathrm{RouteBI}}_{c}\left(t\right) & \equiv {\mathrm{RouteBI}}_{\left(i,d\right)}\left(t\right),\; & {\mathrm{RouteScore}}_{c}\left(t\right) & \equiv {\mathrm{RouteScore}}_{\left(i,d\right)}\left(t\right). \end{array}$$

Route Stability remains a route-level quantity. In AODV, its value is associated with the current local route toward destination *d* through next hop *i*. The primitive BI, NeighborBI, and Trust states remain node-level quantities, whereas destination-dependent aggregation is performed only in RouteBI and RouteScore.

BI and Trust describe different aspects of observed operation and use different memory horizons. Preserving data provenance and event classes enables their correlation to be analyzed without repeatedly treating the same observation as independent evidence.

Table 4 defines the core identifiers and operators that are reused throughout the formal model.

Table 5 specifies the node-, link-, route-, and candidate-level states together with the evidence conditions under which each state is available.

Table 6 isolates candidate-level score, confidence, and gate metadata from the primary node-level state.

Table 7 compares the temporal horizon, source, and memory rule of each current, historical, and candidate-level state.

Table 7 makes the memory boundary explicit: direct Trust and Neighbor Reputation are current aggregates, ${\hat{\mathrm{Trust}}}_{i}$ is the current update target, and only ${\mathrm{Trust}}_{i}$ is the stored historical state in the reference model.

Table 4, Table 5 and Table 6 separate identifiers, node and route states, and candidate metadata so that every availability condition remains readable.

Table 8 completes the notation by defining the adapter, missing-data, decision-epoch, and bounded-update symbols.

#### Rule R-MISSING (Absence of Usable Evidence)

Initialization constants may keep storage and interfaces numerically well formed, but they never constitute evidence. A potentially consumed component is active only when it is current, resolvable, provenance-admissible, confidence-sufficient, and version-compatible:

(4) $$a_{i}^{X}\left(t\right)=⊮\;\left[\begin{array}{c} X\;\mathrm{is}\;\mathrm{current},\;\mathrm{resolvable},\;\mathrm{and}\;\mathrm{provenance}\text{-}\mathrm{admissible},\\ X\;\mathrm{is}\;\mathrm{confidence}\text{-}\mathrm{sufficient}\;\mathrm{and}\;\mathrm{version}\text{-}\mathrm{compatible} \end{array}\right].$$

If $a_{i}^{X}\left(t\right)=0$, the component is absent from both the numerator and denominator. The remaining positive weights are renormalized. An empty active set is undefined. This rule prevents fabricated reward or penalty. It does not, by itself, guarantee equal decision bases across candidates. Candidate comparison therefore uses the adapter-level common-basis and coverage rules defined in Section 4.7 and Section 5.2.1.

### 4.2. Observation Scope and Normalization

Observations concerning a node, link, next hop, or route are collected within a local observation window:

(5) W(t)=[t−Δt,t],Δt>0.

where Δt>0 denotes the duration of the observation period.

All dimensionless quality metrics are restricted to the interval [0,1] by the operator:

(6) C(x)=min{1,max(0,x)}.

A value close to 1 indicates a favorable state, whereas a value close to 0 indicates an unfavorable state. Normalization combines quantities expressed in different units while preserving correlation and common causes of variation.

#### Operational Event Classes and Data Provenance

To limit repeated use of the same observation, TA-BI distinguishes four operational event classes:

- **Local transmission failure**—a failure of a local transmission to a neighbor, used in local packet loss;
- **Unperformed expected forwarding**—failure to perform an expected forwarding operation after successful packet delivery to the neighbor, used in Forwarding Ratio;
- **Local link loss**—a confirmed loss of a local link or neighborhood relation, used in Link Stability;
- **Active route or next-hop change**—a canonical change of the active route or next hop, used in Route Stability.

These events may share a physical cause, but they are not automatically treated as identical or as independent evidence. An observation record should retain at least the event type, observer, assessed object, timestamp, source layer, packet or event identifier, resolution status, and a causal-group identifier whenever the dependency can be established.

When one physical event produces consequences at several layers, separate records require retained provenance and causal relations. Calibration, correlation analysis, and ablation studies then quantify redundancy without assuming orthogonality.

### 4.3. Behavioral Information

Let

(7) $$Y_{1,i}^{\mathrm{BI}}\left(t\right)=Q_{\mathrm{loss},i}\left(t\right),\;Y_{2,i}^{\mathrm{BI}}\left(t\right)=Q_{\mathrm{delay},i}\left(t\right),\;b_{1}^{\mathrm{BI}}=\alpha ,\;b_{2}^{\mathrm{BI}}=\beta ,$$

and define the active component set

(8) $$B_{i}^{\mathrm{BI}}\left(t\right)=\left\{q\in \left\{1,2\right\}:Y_{q,i}^{\mathrm{BI}}\left(t\right)\;\mathrm{is}\;\mathrm{defined},\;\mathrm{current},\;\mathrm{and}\;\mathrm{evidence}\text{-}\mathrm{supported}\right\}.$$

Behavioral Information is then

(9) $${\mathrm{BI}}_{i}\left(t\right)=\left\{\begin{array}{cc} C\;\left[\frac{\sum_{q\in B_{i}^{\mathrm{BI}}\left(t\right)}b_{q}^{\mathrm{BI}}Y_{q,i}^{\mathrm{BI}}\left(t\right)}{\sum_{q\in B_{i}^{\mathrm{BI}}\left(t\right)}b_{q}^{\mathrm{BI}}}\right],\; & \sum_{q\in B_{i}^{\mathrm{BI}}\left(t\right)}b_{q}^{\mathrm{BI}}>0,\\ \mathrm{undefined},\; & \mathrm{otherwise}. \end{array}\right.$$

The configured weights satisfy

(10) α+β=1,α,β≥0.

When both primitive components are available, Equation (9) reduces to the configured weighted sum. With one active component, its normalized weight is one. All other cases follow rule R-MISSING. BI represents the current, locally observable communication quality associated with assessed node *i*, based on local packet loss and delay. Route Stability remains a route-level quantity. In the AODV adapter it is incorporated into RouteBI indexed by (i,d), whereas a full-path adapter incorporates it into the RouteBI of route *r*. BI is a communication-quality descriptor, not a classifier of node intent.

#### 4.3.1. Local Packet Loss

The local packet loss represents the local transmission and link quality; the Forwarding Ratio separately represents cooperative forwarding.

Let $N_{\mathrm{tx},i}\left(t\right)$ denote the number of local transmissions directed to node *i*, and let $N_{\mathrm{fail},i}\left(t\right)$ denote the number of transmissions classified as unsuccessful under the adopted medium access control (MAC)-layer policy, for example, after exhausting the retransmission limit:

(11) $$P_{\mathrm{loss},i}\left(t\right)=\left\{\begin{array}{cc} \frac{N_{\mathrm{fail},i}\left(t\right)}{N_{\mathrm{tx},i}\left(t\right)},\; & N_{\mathrm{tx},i}\left(t\right)>0,\\ \mathrm{undefined},\; & N_{\mathrm{tx},i}\left(t\right)=0. \end{array}\right.$$

(12) $$Q_{\mathrm{loss},i}\left(t\right)=1-P_{\mathrm{loss},i}\left(t\right),\;P_{\mathrm{loss},i}\left(t\right)\;\mathrm{defined}.$$

$P_{\mathrm{loss},0}\in \left[0,1\right]$ may initialize internal storage only. When $N_{\mathrm{tx},i}\left(t\right)=0$, the loss component has availability zero and is omitted from BI.

The local packet loss and Forwarding Ratio use disjoint observation domains. The former concerns attempts to transmit to a neighbor; the latter includes only packets successfully delivered to the neighbor that, according to routing logic, should subsequently be forwarded.

This domain separation limits double attribution of a single failure to both BI and trust, without excluding a common physical cause.

#### 4.3.2. Average Delay

Let $d_{i,k}$ denote a locally observable delay sample associated with communication with node *i*. It may represent the following:

- Local service time measured with a single clock;
- Round-trip time;
- Routing-mechanism response time;
- One-way delay, provided that the clocks are adequately synchronized.

For $N_{D,i}\left(t\right)>0$,

(13) $$D_{\mathrm{avg},i}\left(t\right)=\frac{1}{N_{D,i}\left(t\right)}\sum_{k=1}^{N_{D,i}\left(t\right)}d_{i,k}.$$

A one-way delay derived from different-node timestamps requires explicit clock synchronization. Without that assumption, the implementation uses round-trip time, local service time, or another single-clock quantity.

The normalized delay component is

(14) $$Q_{\mathrm{delay},i}\left(t\right)=\exp\;\left[-{\left(\frac{D_{\mathrm{avg},i}\left(t\right)}{D_{\mathrm{ref}}}\right)}^{\kappa_{D}}\right].$$

where $D_{\mathrm{ref}}>0$ is the reference delay and $\kappa_{D}>0$ controls the sensitivity of the function. Equation (14) is defined only when $N_{D,i}\left(t\right)>0$. If no valid sample is available, $Q_{\mathrm{delay},0}\in \left[0,1\right]$ may initialize internal storage, but the delay component has availability zero and is omitted from BI.

#### 4.3.3. Route Stability

Route Stability is a route-level metric, whereas Link Stability describes one local neighbor connection. In the AODV specialization, the current local route reaches destination *d* through next hop *i*; index (i,d) therefore denotes local Route Stability without requiring the complete node sequence.

(15) $$S_{r}\left(t\right)=1-C\;\left(\frac{R_{\mathrm{changes},r}\left(t\right)}{R_{\max}}\right).$$

For the reference AODV specialization, the generic definition is mapped to the local route toward destination *d* through next hop *i*:

(16) $$S_{i,d}\left(t\right)=1-C\;\left(\frac{R_{\mathrm{changes},i,d}\left(t\right)}{R_{\max}}\right).$$

The pair (i,d) remains the public metric key. The adapter distinguishes successive installations of the same entry by an internal version $\nu_{i,d}$, derived from a sequence number, installation time, or canonical activation event:

(17) $$r_{i,d}^{\left(\nu \right)}\left(t\right)=\left(d,i,\nu_{i,d}\left(t\right)\right).$$

In Equation (15), $R_{\mathrm{changes},r}\left(t\right)$ denotes the number of canonical changes of route *r*. In the specialization in Equation (16), counter $R_{\mathrm{changes},i,d}\left(t\right)$ also increases when version $\nu_{i,d}$ changes, even if next hop *i* and destination *d* remain unchanged. $R_{\max}>0$ is the normalization value and C(x) is the operator defined in Equation (6).

To avoid counting a single reconfiguration process multiple times, route breakage, rediscovery initiation, and activation of a new route are not unconditionally summed as independent changes. The implementation should use one canonical active-route-change counter or mutually exclusive event classes.

The boundary values follow directly from the definition:

(18) $$R_{\mathrm{changes},r}\left(t\right)=0\Rightarrow S_{r}\left(t\right)=1,\;R_{\mathrm{changes},r}\left(t\right)\geq R_{\max}\Rightarrow S_{r}\left(t\right)=0.$$

### 4.4. Neighbor Behavioral Information

Neighbor Behavioral Information aggregates only admissible reports from other nodes concerning observed communication quality or behavior:

(19) $${\mathrm{NeighborBI}}_{i}\left(t\right)=\left\{\begin{array}{cc} \frac{\sum_{j\in N_{i}\left(t\right)}\omega_{j,i}^{\mathrm{BI}}\left(t\right)\;{\hat{\mathrm{BI}}}_{j\to i}\left(t\right)}{\sum_{j\in N_{i}\left(t\right)}\omega_{j,i}^{\mathrm{BI}}\left(t\right)},\; & \sum_{j\in N_{i}\left(t\right)}\omega_{j,i}^{\mathrm{BI}}\left(t\right)>0,\\ \mathrm{undefined},\; & \mathrm{otherwise}. \end{array}\right.$$

The report weight incorporates the reporter’s prior trust, report age, and source admissibility:

(20) $$\omega_{j,i}^{\mathrm{BI}}\left(t\right)={\mathrm{Trust}}_{j}\left(t^{-}\right)\exp\;\left[-\mu_{\mathrm{BI}}\left(t-t_{j,i}^{\mathrm{report}}\right)\right]⊮\;\left[{\mathrm{Trust}}_{j}\left(t^{-}\right)\geq T_{\min}\right]⊮\;\left[\chi_{j,i}^{\mathrm{BI}}\left(t\right)=1\right],$$

where $t^{-}$ denotes the most recent Trust state strictly preceding the current aggregation and $\chi_{j,i}^{\mathrm{BI}}\left(t\right)$ indicates successful provenance, integrity, freshness, and semantic-admissibility checks. Using a preceding Trust state limits direct cyclic dependence between the reporter assessment and the value computed in the current update.

A zero denominator makes NeighborBI unavailable; subsequent aggregation follows rule R-MISSING.

### 4.5. Route Behavioral Information

RouteBI is a candidate-level quantity whose index is supplied by the adapter: (i,d) for the reference AODV adapter and *r* for complete-path adapters. The reference AODV adapter identifies candidate *c* by the next-hop–destination pair (i,d) because AODV exposes a next-hop and routing-table entry rather than the complete forwarding-node sequence. A complete-path adapter identifies the same public abstraction by route identifier *r*. Consequently, the AODV mapping from node state to candidate state is direct:

(21) $$\begin{array}{cccc} {\mathrm{BI}}_{\left(i,d\right)}^{\mathrm{loc}}\left(t\right) & \equiv {\mathrm{BI}}_{i}\left(t\right), & & \\ {\mathrm{NeighborBI}}_{\left(i,d\right)}\left(t\right) & \equiv {\mathrm{NeighborBI}}_{i}\left(t\right),\; & S_{\left(i,d\right)}\left(t\right) & \equiv S_{r_{\left(i,d\right)}}\left(t\right). \end{array}$$

The first component describes local communication quality with the next hop, the second retains controlled indirect information concerning the same node, and the third describes the stability of the specific route toward the destination. When all three components are defined, current, and evidence-supported, the reference RouteBI value is

(22) $${\mathrm{RouteBI}}_{\left(i,d\right)}\left(t\right)=C\;\left[m_{1}{\mathrm{BI}}_{i}\left(t\right)+m_{2}{\mathrm{NeighborBI}}_{i}\left(t\right)+m_{3}S_{\left(i,d\right)}\left(t\right)\right].$$

subject to

(23) $$m_{1}+m_{2}+m_{3}=1,\;m_{1},m_{2},m_{3}\geq 0.$$

Equation (22) is the all-components case. Otherwise, Equation (29) applies rule R-MISSING. The node values remain inputs; the route state $S_{i,d}\left(t\right)$ and pair key (i,d) make the resulting RouteBI candidate-specific.

For a complete route *r*, let $V\left(r\right)=\left\{v_{1},\ldots ,v_{h_{r}}\right\}$ be the required forwarding-node sequence. The source and destination are excluded in the reference profile unless the adapter declares them observable. For X∈{BI,NBI}, define

(24) $$V_{\mathrm{valid}}^{X}\left(r,t\right)=\left\{v_{k}\in V\left(r\right):a_{v_{k}}^{X}\left(t\right)=1,\;\Delta t_{v_{k}}^{X}\left(t\right)\leq \tau_{X},\;{\mathrm{Conf}}_{v_{k}}^{X}\left(t\right)\geq {\mathrm{Conf}}_{\min}^{X}\right\}.$$

With non-negative node weights $\eta_{k}$ and a positive total required weight, path-component coverage is

(25) $${\mathrm{Comp}}_{r}^{X}\left(t\right)=\frac{\sum_{v_{k}\in V_{\mathrm{valid}}^{X}\left(r,t\right)}\eta_{k}}{\sum_{v_{k}\in V\left(r\right)}\eta_{k}},\;X\in \left\{\mathrm{BI},\mathrm{NBI}\right\}.$$

A partial aggregate may be retained for diagnostics,

(26) $$X_{r}^{\mathrm{diag}}\left(t\right)=\frac{\sum_{v_{k}\in V_{\mathrm{valid}}^{X}\left(r,t\right)}\eta_{k}X_{v_{k}}\left(t\right)}{\sum_{v_{k}\in V_{\mathrm{valid}}^{X}\left(r,t\right)}\eta_{k}},$$

when the denominator is positive. The canonical complete-path adapter exposes $X_{r}\left(t\right)=X_{r}^{\mathrm{diag}}\left(t\right)$ for decision use only when ${\mathrm{Comp}}_{r}^{X}\left(t\right)=1$. Otherwise, $X_{r}\left(t\right)$ is undefined. Thus, removal of an unavailable weak node cannot improve a decision-usable route component.

For AODV, the candidate components remain

(27) $$Z_{1,c}\left(t\right)={\mathrm{BI}}_{c}^{\mathrm{loc}}\left(t\right),\;Z_{2,c}\left(t\right)={\mathrm{NeighborBI}}_{c}\left(t\right),\;Z_{3,c}\left(t\right)=S_{c}\left(t\right),$$

with active set

(28) $$A_{c}\left(t\right)=\left\{q\in \left\{1,2,3\right\}:Z_{q,c}\left(t\right)\;\mathrm{is}\;\mathrm{defined},\;\mathrm{current},\;\mathrm{and}\;\mathrm{evidence}\text{-}\mathrm{supported}\right\}.$$

The candidate-level diagnostic RouteBI operator is

(29) $${\mathrm{RouteBI}}_{c}\left(t\right)=\left\{\begin{array}{cc} C\;\left[\frac{\sum_{q\in A_{c}\left(t\right)}m_{q}Z_{q,c}\left(t\right)}{\sum_{q\in A_{c}\left(t\right)}m_{q}}\right], & \sum_{q\in A_{c}\left(t\right)}m_{q}>0,\\ \mathrm{undefined}, & \mathrm{otherwise}. \end{array}\right.$$

When every component is available, Equation (29) reduces to the all-components AODV or complete-path mapping. A partial value is not automatically eligible for cross-candidate comparison; the adapter applies a common mask before RouteScore influences AODV ordering.

### 4.6. Neighbor Reputation and Trust

For a direct assessment produced by observer *j*, let

(30) $$Y_{1,j\to i}^{\mathrm{dir}}\left(t\right)={\mathrm{FR}}_{j\to i}\left(t\right),\;Y_{2,j\to i}^{\mathrm{dir}}\left(t\right)={\mathrm{LS}}_{j\to i}\left(t\right),$$

with non-negative weights $a_{1},a_{2}$ and active set

(31) $$D_{j\to i}\left(t\right)=\left\{q\in \left\{1,2\right\}:Y_{q,j\to i}^{\mathrm{dir}}\left(t\right)\;\mathrm{is}\;\mathrm{defined},\;\mathrm{current},\;\mathrm{and}\;\mathrm{evidence}\text{-}\mathrm{supported}\right\}.$$

The direct assessment excludes indirect reputation and is

(32) $${\mathrm{Trust}}_{j\to i}^{\mathrm{direct}}\left(t\right)=\left\{\begin{array}{cc} \frac{\sum_{q\in D_{j\to i}\left(t\right)}a_{q}Y_{q,j\to i}^{\mathrm{dir}}\left(t\right)}{\sum_{q\in D_{j\to i}\left(t\right)}a_{q}},\; & \sum_{q\in D_{j\to i}\left(t\right)}a_{q}>0,\\ \mathrm{undefined},\; & \mathrm{otherwise}. \end{array}\right.$$

Neighbor Reputation is a weighted aggregation of defined direct assessments:

(33) $${\mathrm{NR}}_{i}\left(t\right)=\left\{\begin{array}{cc} \frac{\sum_{j\in R_{i}\left(t\right)}\rho_{j,i}\left(t\right)\;{\mathrm{Trust}}_{j\to i}^{\mathrm{direct}}\left(t\right)}{\sum_{j\in R_{i}\left(t\right)}\rho_{j,i}\left(t\right)},\; & \sum_{j\in R_{i}\left(t\right)}\rho_{j,i}\left(t\right)>0,\\ \mathrm{undefined},\; & \mathrm{otherwise}. \end{array}\right.$$

The reporter weight is explicitly defined by

(34) $$\rho_{j,i}\left(t\right)={\mathrm{Trust}}_{j}\left(t^{-}\right)\exp\;\left[-\mu_{\mathrm{NR}}\left(t-t_{j,i}^{\mathrm{report}}\right)\right]⊮\;\left[{\mathrm{Trust}}_{j}\left(t^{-}\right)\geq T_{\min}\right]⊮\;\left[\chi_{j,i}^{\mathrm{NR}}\left(t\right)=1\right],$$

where $\chi_{j,i}^{\mathrm{NR}}\left(t\right)$ represents provenance, integrity, freshness, and semantic-admissibility checks. Consequently, $\rho_{j,i}\left(t\right)\in \left[0,1\right]$ when ${\mathrm{Trust}}_{j}\in \left[0,1\right]$.

For the node’s raw Trust estimate, define

(35) $$Y_{1,i}^{\mathrm{Tr}}\left(t\right)={\mathrm{FR}}_{i}\left(t\right),\;Y_{2,i}^{\mathrm{Tr}}\left(t\right)={\mathrm{LS}}_{i}\left(t\right),\;Y_{3,i}^{\mathrm{Tr}}\left(t\right)={\mathrm{NR}}_{i}\left(t\right),$$

and

(36) $$B_{i}^{\mathrm{Tr}}\left(t\right)=\left\{q\in \left\{1,2,3\right\}:Y_{q,i}^{\mathrm{Tr}}\left(t\right)\;\mathrm{is}\;\mathrm{defined},\;\mathrm{current},\;\mathrm{and}\;\mathrm{evidence}\text{-}\mathrm{supported}\right\}.$$

The raw Trust value is

(37) $${\mathrm{Trust}}_{i}^{\mathrm{raw}}\left(t\right)=\left\{\begin{array}{cc} C\;\left[\frac{\sum_{q\in B_{i}^{\mathrm{Tr}}\left(t\right)}w_{q}Y_{q,i}^{\mathrm{Tr}}\left(t\right)}{\sum_{q\in B_{i}^{\mathrm{Tr}}\left(t\right)}w_{q}}\right],\; & \sum_{q\in B_{i}^{\mathrm{Tr}}\left(t\right)}w_{q}>0,\\ \mathrm{undefined},\; & \mathrm{otherwise}, \end{array}\right.$$

subject to

(38) $$w_{1}+w_{2}+w_{3}=1,\;w_{1},w_{2},w_{3}\geq 0.$$

Let $t_{k}$ be the *k*-th local update instant. The evidence-supported Trust state is updated only when the raw estimate is defined:

(39) $${\mathrm{Trust}}_{i,k}=\left\{\begin{array}{cc} C\;\left[\left(1-\delta_{T,i,k}\right){\mathrm{Trust}}_{i,k-1}+\delta_{T,i,k}{\mathrm{Trust}}_{i}^{\mathrm{raw}}\left(t_{k}\right)\right],\; & {\mathrm{Trust}}_{i}^{\mathrm{raw}}\left(t_{k}\right)\;\mathrm{defined},\\ {\mathrm{Trust}}_{i,k-1},\; & \mathrm{otherwise}. \end{array}\right.$$

When no current raw estimate exists, the numerical Trust state is held and its age increases. Its availability is then determined by rule R-MISSING; an initialization value cannot enter the raw Trust aggregation as evidence. NeighborBI aggregates reported communication quality or behavior, while Neighbor Reputation aggregates direct cooperativeness assessments from other observers. Provenance controls reuse of one observation across BI, NeighborBI, direct Trust, and Neighbor Reputation.

#### 4.6.1. Forwarding Ratio

The Forwarding Ratio represents execution of an expected forwarding operation after the packet has been successfully received by the assessed node.

(40) $${\hat{\mathrm{FR}}}_{i}\left(t_{k}\right)=\left\{\begin{array}{cc} \frac{N_{\mathrm{fwd},i}\left(t_{k}\right)}{N_{\exp,i}\left(t_{k}\right)},\; & N_{\exp,i}\left(t_{k}\right)>0,\\ \mathrm{undefined},\; & N_{\exp,i}\left(t_{k}\right)=0. \end{array}\right.$$

where $N_{\exp,i}\left(t_{k}\right)$ is the number of packets successfully delivered to node *i* that should subsequently be forwarded, and $N_{\mathrm{fwd},i}\left(t_{k}\right)$ is the number of those packets whose correct forwarding was resolved and observed.

The temporally stored state is

(41) $${\mathrm{FR}}_{i,k}=\left\{\begin{array}{cc} C\;\left[\left(1-\delta_{\mathrm{FR},i,k}\right){\mathrm{FR}}_{i,k-1}+\delta_{\mathrm{FR},i,k}{\hat{\mathrm{FR}}}_{i}\left(t_{k}\right)\right],\; & {\hat{\mathrm{FR}}}_{i}\left(t_{k}\right)\;\mathrm{defined},\\ {\mathrm{FR}}_{i,k-1},\; & \mathrm{otherwise}. \end{array}\right.$$

A window with $N_{\exp,i}=0$ contributes no forwarding evidence. The stored FR value is held, aged, and exposed only when rule R-MISSING remains satisfied.

#### 4.6.2. Link Stability

Link Stability describes the availability and persistence of a single local connection to a neighbor:

(42) $$\nu_{\mathrm{break},i}\left(t\right)=\frac{B_{i}\left(t\right)}{\Delta t},\;{\mathrm{LS}}_{i}\left(t\right)=\exp\;\left[-\nu_{\mathrm{break},i}\left(t\right)\tau_{\mathrm{ref}}\right].$$

where $B_{i}\left(t\right)$ denotes the number of identified local-link failures and $\tau_{\mathrm{ref}}>0$ is the reference time constant.

### 4.7. RouteScore and Evidence Completeness

At adapter level, *c* denotes a generic routing candidate. Candidate Trust is derived from node-level Trust without creating destination-specific Trust copies. For the AODV candidate (i,d), ${\mathrm{Trust}}_{c}\left(t\right)={\mathrm{Trust}}_{i}\left(t\right)$ when the node state is current and admissible.

For a complete route, let V(c) be the required forwarding-node set and let $\xi_{v}>0$ be declared coverage weights. Define

(43) $$V_{\mathrm{valid}}^{\mathrm{Tr}}\left(c,t\right)=\left\{v\in V\left(c\right):a_{v}^{\mathrm{Tr}}\left(t\right)=1,\;\Delta t_{v}^{\mathrm{Tr}}\left(t\right)\leq \tau_{\mathrm{Tr}},\;{\mathrm{Conf}}_{v}^{\mathrm{Tr}}\left(t\right)\geq {\mathrm{Conf}}_{\min}^{\mathrm{Tr}}\right\},$$

(44) $${\mathrm{Comp}}_{c}^{\mathrm{Tr}}\left(t\right)=\frac{\sum_{v\in V_{\mathrm{valid}}^{\mathrm{Tr}}\left(c,t\right)}\xi_{v}}{\sum_{v\in V(c)}\xi_{v}}.$$

The canonical weakest-link value is decision-usable only with full coverage:

(45) $${\mathrm{Trust}}_{c}\left(t\right)=\left\{\begin{array}{cc} \min_{v\in V(c)}{\mathrm{Trust}}_{v}\left(t\right), & {\mathrm{Comp}}_{c}^{\mathrm{Tr}}\left(t\right)=1,\\ \mathrm{undefined}, & {\mathrm{Comp}}_{c}^{\mathrm{Tr}}\left(t\right)<1. \end{array}\right.$$

A partial minimum may be logged as a diagnostic quantity, but it cannot open the decision gate. This rule prevents an unavailable low-Trust node from disappearing from the weakest-link calculation.

For q∈{1,2}, define

(46) $$Y_{1,c}^{\mathrm{RS}}\left(t\right)={\mathrm{RouteBI}}_{c}\left(t\right),\;Y_{2,c}^{\mathrm{RS}}\left(t\right)={\mathrm{Trust}}_{c}\left(t\right),\;\lambda_{1}=\lambda_{\mathrm{BI}},\;\lambda_{2}=\lambda_{\mathrm{Tr}},$$

where $\lambda_{\mathrm{BI}}+\lambda_{\mathrm{Tr}}=1$ and both coefficients are non-negative. Component confidence is

(47) $${\mathrm{Conf}}_{c}^{q}\left(t\right)=\left\{\begin{array}{cc} \min\;\left(1,\frac{N_{c,q}^{\mathrm{obs}}\left(t\right)}{N_{\min,q}}\right)\exp\left[-\mu_{q}\left(t-t_{c,q}^{\mathrm{last}}\right)\right], & \begin{array}{c} \lambda_{q}>0,\;Y_{q,c}^{\mathrm{RS}}\left(t\right)\;\mathrm{defined},\\ Y_{q,c}^{\mathrm{RS}}\left(t\right)\;\mathrm{current}\;\mathrm{and}\;\mathrm{admissible},\\ N_{c,q}^{\mathrm{obs}}\left(t\right)>0 \end{array}\\ 0, & \mathrm{otherwise}. \end{array}\right.$$

The candidate diagnostic active set is

(48) $$B_{c}^{\mathrm{RS}}\left(t\right)=\left\{q\in \left\{1,2\right\}:\lambda_{q}>0,\;Y_{q,c}^{\mathrm{RS}}\left(t\right)\;\mathrm{is}\;\mathrm{current}\;\mathrm{and}\;\mathrm{admissible}\right\}.$$

A local diagnostic score may use active-weight renormalization:

(49) $${\mathrm{RouteScore}}_{c}^{\mathrm{diag}}\left(t\right)=\left\{\begin{array}{cc} C\;\left[\frac{\sum_{q\in B_{c}^{\mathrm{RS}}\left(t\right)}\lambda_{q}Y_{q,c}^{\mathrm{RS}}\left(t\right)}{\sum_{q\in B_{c}^{\mathrm{RS}}\left(t\right)}\lambda_{q}}\right], & \sum_{q\in B_{c}^{\mathrm{RS}}\left(t\right)}\lambda_{q}>0,\\ \mathrm{undefined}, & \mathrm{otherwise}. \end{array}\right.$$

Its candidate-level completeness is

(50) $${\mathrm{Comp}}_{c}^{\mathrm{RS}}\left(t\right)=\frac{\sum_{q\in B_{c}^{\mathrm{RS}}\left(t\right)}\lambda_{q}}{\sum_{q\in \left\{1,2\right\}:\lambda_{q}>0}\lambda_{q}}.$$

The metadata above describe one candidate. They do not authorize comparison of candidates built from different component sets. Every adapter must define a comparison basis. The canonical AODV basis is the common full mask in Section 5.2.1. When both components are available, the score reduces to

(51) $${\mathrm{RouteScore}}_{\left(i,d\right)}\left(t\right)=C\left[\lambda_{\mathrm{BI}}{\mathrm{RouteBI}}_{\left(i,d\right)}\left(t\right)+\lambda_{\mathrm{Tr}}{\mathrm{Trust}}_{i}\left(t\right)\right].$$

The Route Decision Engine supplies typed component values and metadata. The protocol adapter forms $C_{\mathrm{feas}}\left(t\right)$, selects the comparison basis, and applies

(52) $$K_{c}^{A}\left(t\right)=\Phi_{A}\;\left(U_{c}^{\mathrm{base}}\left(t\right),\Gamma_{A}\left(t\right),{\mathrm{RouteScore}}_{c}^{A}\left(t\right)\right),\;c\in C_{\mathrm{feas}}\left(t\right),$$

(53) $$c^{*}\left(t\right)\in \arg\max_{c\in C_{\mathrm{feas}}\left(t\right)}^{{≻}_{A}}K_{c}^{A}\left(t\right).$$

A closed comparison gate invokes the baseline key:

(54) $$\Phi_{A}\;\left(U_{c}^{\mathrm{base}}\left(t\right),0,{\mathrm{RouteScore}}_{c}^{A}\left(t\right)\right)\equiv \Phi_{A}^{\mathrm{base}}\;\left(U_{c}^{\mathrm{base}}\left(t\right)\right).$$

The candidate remains feasible under native rules; it receives neither a fabricated neutral score nor a missing-data penalty.

### 4.8. Execution Semantics, Versioning, and Atomicity

A conformant implementation exposes typed records rather than unstructured scalar values. The minimum logical types are

(55) $$\begin{array}{cc} {\mathrm{NodeState}}_{i} & =({\mathrm{BI}}_{i},{\mathrm{NeighborBI}}_{i},{\mathrm{FR}}_{i},{\mathrm{LS}}_{i},{\mathrm{NR}}_{i},{\hat{\mathrm{Trust}}}_{i},{\mathrm{Trust}}_{i},a_{i},{\mathrm{Conf}}_{i},t_{i}),\\ {\mathrm{CandidateState}}_{i,d}^{\nu } & =(S_{i,d}^{\nu },{\mathrm{RouteBI}}_{i,d}^{\nu },{\mathrm{Trust}}_{i,d}^{\nu },{\mathrm{RouteScore}}_{i,d}^{\nu },{\mathrm{Conf}}_{i,d}^{\nu },g_{i,d}^{\nu }), \end{array}$$

where $a_{i}$, ${\mathrm{Conf}}_{i}$, and $t_{i}$ collect component availability, confidence, and evidence timestamps. The adapter result type is

(56) $${\mathrm{Decision}}_{A}=\mathrm{Select}\left(c\right)\;|\;\mathrm{Fallback}\left(r_{f}\right),$$

where $r_{f}$ identifies the native reason, such as an empty feasible set, closed evidence gate, failed commit-time revalidation, or exhaustion of the frozen order.

Each local event *e* receives a key,

(57) $$\kappa \left(e\right)=\left(t_{e},p_{e},s_{e}\right),$$

ordered lexicographically by physical time $t_{e}$, event priority $p_{e}$, and a monotone local serial number $s_{e}$. The equal-time priorities are as follows: (1) native route invalidation or route-instance change; (2) observation-window closure; (3) direct or indirect evidence update; (4) RREP admission; and (5) epoch closure and route commit. This order ensures that a route change invalidates old candidate state before a same-time Trust or RouteScore calculation, while an RREP stamped at the closing time is admitted before the epoch is frozen.

Closing window $W_{k}$ is atomic with respect to its event key. Records with $\kappa \left(e\right)\leq \left(t_{k},2,\infty \right)$ are included in the immutable snapshot for $W_{k}$; later records enter $W_{k+1}$. Node-state publication and candidate-state publication are each atomic writes. The adapter reads one candidate snapshot per version and revalidates the native route-table entry after ranking but before installation. A route event that occurs between ranking and commit therefore causes revalidation failure rather than a mixed-version decision.

When a route change and a Trust update share a timestamp, the route version is advanced first. The node-level Trust update remains valid because it is keyed by *i*, whereas all candidate-level values computed for the previous $\nu_{i,d}$ become unavailable. When epoch closure and an RREP arrival share a timestamp, the RREP is processed first under the stated priority and is either admitted or rejected by native AODV rules before the set is frozen.

### 4.9. Locally Coordinated Common Bounded Update Rule

The common bounded update applies to states with declared historical memory. The canonical baseline uses it for Forwarding Ratio and Trust; BI and NeighborBI represent the current horizon, Link and Route Stability derive from event windows, and RouteScore remains unfiltered.

Let $t_{k}$ denote the physical time of the *k*-th local update and $\Delta t_{k}=t_{k}-t_{k-1}>0$. Define $X_{i,k}=X_{i}\left(t_{k}\right)$ and ${\hat{X}}_{i,k}={\hat{X}}_{i}\left(t_{k}\right)$. The canonical recursion is

(58) $$X_{i,k}=\left(1-\delta_{X,i,k}\right)X_{i,k-1}+\delta_{X,i,k}{\hat{X}}_{i,k},$$

with

(59) $$0<\delta_{X,\min}\leq \delta_{X,i,k}\leq \delta_{X,\max}\leq 1.$$

For the canonical baseline, X∈{FR,Trust}. A state is updated by Equation (58) only when ${\hat{X}}_{i,k}$ is defined; otherwise its numerical value is held and its evidence age increases.

For a constant coefficient satisfying $0<\delta_{X}<1$, the half-life of the previous state’s influence, measured in update steps, is

(60) $$H_{X}^{\mathrm{steps}}=\frac{\ln(0.5)}{\ln\left(1-\delta_{X}\right)}.$$

For constant sampling interval Δt, the corresponding physical half-life is

(61) $$T_{1/2,X}=H_{X}^{\mathrm{steps}}\;\Delta t.$$

Conversely,

(62) $$\delta_{X}=1-2^{-1/H_{X}^{\mathrm{steps}}},\;H_{X}^{\mathrm{steps}}>0.$$

The boundary case $\delta_{X}=1$ is the memoryless update $X_{i,k}={\hat{X}}_{i,k}$ and bypasses Equation (60), whose logarithm is undefined at that boundary.

An adaptive variant may use normalized observation variability and current-data reliability:

(63) $$\delta_{X,i,k}=\delta_{X,\min}+\left(\delta_{X,\max}-\delta_{X,\min}\right)Q_{i,k}A_{X,i,k},$$

where

(64) $$A_{X,i,k}\in \left[0,1\right],\;Q_{i,k}\in \left[0,1\right],$$

and

(65) $$Q_{i,k}=\min\;\left\{1,\frac{N_{i,k}^{\mathrm{obs}}}{N_{\min}}\right\}\exp\;\left[-\mu \left(t_{k}-t_{i}^{\mathrm{last}}\right)\right].$$

The domains in Equation (64) guarantee $\delta_{X,i,k}\in \left[\delta_{X,\min},\delta_{X,\max}\right]$. The adaptive coefficient is a bounded heuristic parameter, not a learned model.

Section 6 states the range-preservation and conditional-convergence results for Equation (58). It also analyzes AODV hysteresis on the third component of the actual lexicographic key and gives a sufficient condition that excludes an immediate reverse switch.

### 4.10. Parameter Admissibility and Analytical Sensitivity

The formal architecture distinguishes admissibility constraints from empirical calibration. Let Θ denote the complete parameter vector. Its admissible domain $\Omega_{\Theta }$ requires: non-negative aggregation weights; a positive sum for every active aggregation; positive normalization and time constants; $0<\delta_{X,\min}\leq \delta_{X,\max}\leq 1$; confidence thresholds in [0,1]; component full-confidence sample scales $N_{\min,q}>0$; $N_{\min}>0$ where the optional adaptive-update reliability term is used; $T_{\mathrm{RREP}}>0$; and $\varepsilon_{\mathrm{sw}}\in \left[0,1\right]$. Parameters that violate these conditions are outside the model.

The parameters have distinct formal roles. BI weights combine local packet loss and delay. RouteBI weights combine local BI, NeighborBI, and Route Stability at the candidate level. Trust weights combine the Forwarding Ratio, Link Stability, and Neighbor Reputation. RouteScore coefficients combine candidate RouteBI and candidate Trust. Update coefficients determine historical memory, confidence parameters determine evidence usability, and adapter parameters determine collection timing and switching margins.

Table 9 separates mathematical admissibility from implementation choice and empirical calibration. The values are deliberately not presented as universal WSN/MWSN defaults. A deployment must derive time constants from observation and route-lifecycle scales, select confidence and sample thresholds from instrumentation reliability, and register all nominal values and sensitivity ranges before performance evaluation.

For every $\Theta \in \Omega_{\Theta }$, the range, missing-evidence, and candidate-admissibility propositions remain valid. Numerical parameter selection is therefore an external identification problem rather than part of the existence or coherence proof. For a fixed active set and fixed normalized weights, Proposition 2 establishes nonexpansiveness in the infinity norm. When normalized weights vary, Equation (82) additionally bounds their contribution in the $l_{1}$ norm. Any deployment-level sensitivity analysis intended to support network-performance inference must vary the weights, ${\mathrm{Conf}}_{\min}$, age limits, $T_{\mathrm{RREP}}$, $\varepsilon_{\mathrm{sw}}$, and update coefficients over preregistered admissible ranges and report both network outcomes and gate activation. The numerical values used by the deterministic oracle are constructed branch witnesses, not optimized or deployment-calibrated WSN/MWSN parameters. Changes in the active set, threshold crossings, or protocol-feasibility transitions are discrete boundary events and are handled by the declared availability and fallback rules.

## 5. Routing-Level Integration Principles

### 5.1. Integration Conditions and Decision Interface

Integration requires at least two candidates admitted by the routing protocol and a decision point at which they can be compared. The adapter forms the feasible set and maps each candidate to the formal TA-BI state; the Route Decision Engine computes RouteScore; the adapter then applies the protocol-specific ordering and revalidates feasibility before execution.

**Figure 5:**
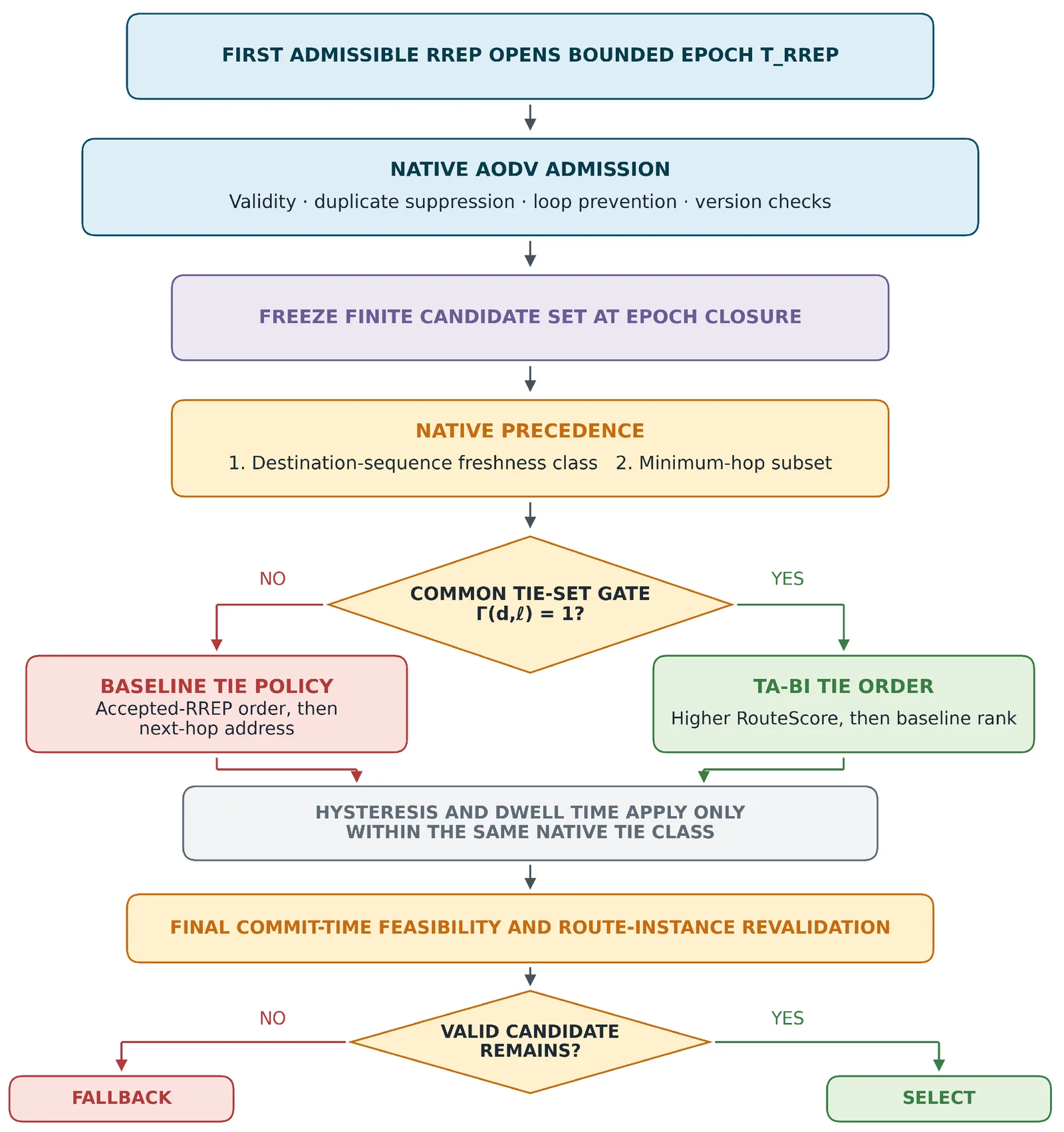
Reference AODV candidate-admission and decision flow supported by TA-BI. RREP denotes route reply and $\Gamma_{d,l}$ is the complete common-basis gate. Native sequence freshness and hop-count precedence remain authoritative.

The reference flow for integrating TA-BI with AODV is shown in Figure 5.

RouteScore is used only after candidates pass the declared native validity, freshness, loop-prevention, feasibility, and confidence checks.

### 5.2. Integration with AODV

AODV is the reference implementation case because RFC 3561 explicitly defines route discovery and repair, destination sequence numbers, route-table entries, hop count, next hop, route lifetime, and route-update rules [3]. The adapter first applies these native validity, freshness, and loop-prevention requirements and then compares only the candidates that remain admissible.

The AODV adapter represents a candidate by the public pair (i,d), where *i* is the next hop and *d* is the destination of the routing entry. Internal version $\nu_{i,d}$ distinguishes successive installations of the same entry. Node-level states are stored once per assessed node, whereas Route Stability, RouteBI, and RouteScore retain the destination-dependent candidate context (i,d).

#### 5.2.1. Reference AODV Decision Epoch, Common Basis, and Candidate Set

The reference adapter uses a conservative lexicographic policy. For route-discovery instance *ℓ* and destination *d*, the first admissible RREP opens

(66) $$I_{d,l}^{\mathrm{RREP}}=\left[t_{d,l}^{\mathrm{open}},t_{d,l}^{\mathrm{close}}\right],\;t_{d,l}^{\mathrm{close}}=t_{d,l}^{\mathrm{open}}+T_{\mathrm{RREP}},\;T_{\mathrm{RREP}}>0.$$

The configured duration cannot exceed the native discovery deadline. RREPs enter $C_{d,l}^{\mathrm{RREP}}$ only after native validity, duplicate, freshness, and loop-prevention checks. The set is frozen at epoch closure.

RFC 3561 freshness is represented by signed 32-bit subtraction:

(67) $$s_{1}{≻}_{\mathrm{seq}}s_{2}⟺\mathrm{int}32\left(\left(s_{1}-s_{2}\right)\;\mathrm{mod}\;2^{32}\right)>0.$$

Under A4, the freshest subset and minimum-hop tie class are

(68) $$C_{d,l}^{\mathrm{seq}}=\left\{c\in C_{d,l}^{\mathrm{RREP}}:∄c^{\prime }\in C_{d,l}^{\mathrm{RREP}}\;\mathrm{with}\;{\mathrm{Seq}}_{c^{\prime }}{≻}_{\mathrm{seq}}{\mathrm{Seq}}_{c}\right\},$$

(69) $$h_{d,l}^{\min}=\min_{c\in C_{d,l}^{\mathrm{seq}}}h_{c},\;C_{d,l}^{\mathrm{tie}}=\left\{c\in C_{d,l}^{\mathrm{seq}}:h_{c}=h_{d,l}^{\min}\right\}.$$

Candidate-specific renormalization is not used to compare this class. The common component mask is

(70) $$B_{d,l}^{\mathrm{cmp}}\left(t\right)=\underset{c\in C_{d,l}^{\mathrm{tie}}}{⋂}B_{c}^{\mathrm{RS}}\left(t\right).$$

Its configured-basis completeness is

(71) $${\mathrm{Comp}}_{d,l}^{\mathrm{cmp}}\left(t\right)=\frac{\sum_{q\in B_{d,l}^{\mathrm{cmp}}\left(t\right)}\lambda_{q}}{\sum_{q\in \left\{1,2\right\}:\lambda_{q}>0}\lambda_{q}}.$$

For each tied candidate, the common-basis score is

(72) $${\mathrm{RouteScore}}_{c}^{\mathrm{cmp}}\left(t\right)=C\;\left[\frac{\sum_{q\in B_{d,l}^{\mathrm{cmp}}\left(t\right)}\lambda_{q}Y_{q,c}^{\mathrm{RS}}\left(t\right)}{\sum_{q\in B_{d,l}^{\mathrm{cmp}}\left(t\right)}\lambda_{q}}\right]$$

when the denominator is positive. The canonical common gate is

(73) $$\Gamma_{d,l}\left(t\right)=⊮\;\left[C_{d,l}^{\mathrm{tie}}\neq ⌀,\;{\mathrm{Comp}}_{d,l}^{\mathrm{cmp}}\left(t\right)=1,\;\min_{\begin{array}{c} c\in C_{d,l}^{\mathrm{tie}}\\ q:\lambda_{q}>0 \end{array}}{\mathrm{Conf}}_{c}^{q}\left(t\right)\geq {\mathrm{Conf}}_{\min}\right].$$

Thus, one missing positively weighted component for one tied candidate closes the gate for the complete tie class. All compared candidates either use the same complete basis or none uses RouteScore. A noncanonical adapter may compare a declared partial common mask, but it cannot claim baseline equivalence solely from rule R-MISSING.

Routing-decision availability and TA-BI enhancement availability are distinct. Under the snapshot semantics of Algorithm 1 and the preconditions of Proposition 7, Algorithm 2 returns either Select(c) or $\mathrm{Fallback}(r_{f})$, so

(74) $$A_{d,l}^{\mathrm{route}}=1,\;A_{d,l}^{\mathrm{TA}\text{-}\mathrm{BI}}=\Gamma_{d,l}\left(t_{d,l}^{\mathrm{close}}\right).$$

The first quantity records the existence of a total adapter outcome. The second records only whether RouteScore is eligible to influence ordering inside the declared native tie class; it neither asserts that a route exists nor measures a network-performance benefit.

For a finite reported set E of route-discovery epochs, the gate-activation ratio is

(75) $${\hat{\rho }}^{\Gamma }\left(E\right)=\frac{1}{\left|E\right|}\sum_{(d,l)\in E}\Gamma_{d,l}\left(t_{d,l}^{\mathrm{close}}\right).$$

Any empirical report of ${\hat{\rho }}^{\Gamma }$ must declare topology, node and mobility roles, traffic, observation windows, age limits, ${\mathrm{Conf}}_{\min}$, $T_{\mathrm{RREP}}$, radio/MAC configuration, seeds, and uncertainty. The quantity is defined but not estimated here.

A deterministic baseline rank $\pi_{c}^{\mathrm{base}}\in N$ is assigned first by accepted-RREP event order and then by next-hop address. The AODV mapping and key are

(76) $$\Phi_{\mathrm{AODV}}\left(-h_{c},{\mathrm{RouteScore}}_{c}^{\mathrm{cmp}},-\pi_{c}^{\mathrm{base}};\Gamma_{d,l}\right)=\left(-h_{c},\Gamma_{d,l}{\mathrm{RouteScore}}_{c}^{\mathrm{cmp}},-\pi_{c}^{\mathrm{base}}\right),$$

(77) $$K_{c,d,l}^{\mathrm{AODV}}=\left({\mathrm{Seq}}_{c},-h_{c},\Gamma_{d,l}{\mathrm{RouteScore}}_{c}^{\mathrm{cmp}},-\pi_{c}^{\mathrm{base}}\right),$$

with

(78) $${≻}_{\mathrm{AODV}}^{\mathrm{lex}}=\mathrm{lex}\left({≻}_{\mathrm{seq}},>,>,>\right).$$

The provisional winner is

(79) $$c_{d,l}^{\mathrm{pre}}=\arg\max_{c\in C_{d,l}^{\mathrm{RREP}}}^{{≻}_{\mathrm{AODV}}^{\mathrm{lex}}}K_{c,d,l}^{\mathrm{AODV}}.$$

For an open gate, RouteScore distinguishes only candidates with equal freshness and hop count. For a closed gate, the score component is identical and the declared baseline rank decides the tie.
**Algorithm 1** Versioned Local TA-BI State-Update Cycle1:**Precondition:** Parameters belong to $\Omega_{\Theta }$; the event set for the closing window is finite; every event carries κ(e), provenance, resolution status, and assessed object; the native route table exposes the current version $\nu_{i,d}$.2:Process equal-time native route invalidations and route-instance changes first. Advance $\nu_{i,d}$ when required and set every mismatched candidate-dependent record to unavailable.3:Atomically close $W_{k}$ and freeze all records with keys admitted by Equation (57). Records outside the snapshot remain in the next window.4:Compute primitive loss, delay, forwarding, link, and route-change observations and their availability, age, and confidence metadata. Unresolved observations remain inactive.5:Compute current BI, NeighborBI, direct Trust, Neighbor Reputation, and raw Trust from their active sets. Remove inactive weights from numerator and denominator and renormalize the remaining positive weights.6:Update only historical states whose current target is defined. Apply Equation (58) to Forwarding Ratio and stored Trust in the reference configuration; otherwise hold the numerical value and increase evidence age.7:Map node state to each current candidate version, compute Route Stability, RouteBI, candidate Trust, RouteScore, basis completeness, confidence, and gate, and reject any input whose version differs from the route-table version.8:Atomically publish one ${\mathrm{NodeState}}_{i}$ record and one ${\mathrm{CandidateState}}_{i,d}^{\nu }$ record per current candidate instance.9:**Postcondition:** Every published scalar is either defined in [0,1] with current metadata or marked unavailable; no candidate state mixes versions; the adapter reads one immutable snapshot.

**Algorithm 2** Total reference AODV adapter decision
  1:**Precondition:** The discovery instance (d,l) has finite input; accepted sequence numbers satisfy A4; $\pi_{c}^{\mathrm{base}}$ is total; candidate snapshots are version-consistent.  2:Admit RREPs during $I_{d,l}^{\mathrm{RREP}}$ only after native AODV validity, duplicate, freshness, and loop-prevention checks.  3:Freeze the set at epoch closure. Return native fallback if it is empty.  4:Form the freshness-maximal class and minimum-hop tie class.  5:Intersect candidate component masks. Compute ${\mathrm{Comp}}_{d,l}^{\mathrm{cmp}}$ and $\Gamma_{d,l}$. An incomplete common basis closes the gate.  6:If the gate is open, compute every score on the same complete mask and compare score followed by baseline rank. If closed, compare baseline rank only.  7:Apply hysteresis and dwell time only inside the same native tie class.  8:Revalidate candidates in frozen order immediately before commit. Return Select(c) for the first feasible and version-valid candidate.  9:If all candidates fail, return native fallback. A strictly fresher late RREP may start a new epoch; other late responses cannot reopen the frozen epoch.10:**Postcondition:** Return one Select or Fallback. Use one common RouteScore mask for every score comparison.


#### 5.2.2. Hysteresis, Late RREPs, and Final Revalidation

If the currently installed candidate $c_{\mathrm{cur}}$ remains feasible and belongs to $C_{d,l}^{\mathrm{tie}}$, a TA-BI-induced switch to the highest-ranked challenger $c_{\mathrm{new}}\in C_{d,l}^{\mathrm{tie}}$ is permitted only when

(80) $$\Gamma_{d,l}=1,\;{\mathrm{RouteScore}}_{c_{\mathrm{new}}}\left(t_{d,l}^{\mathrm{close}}\right)\geq {\mathrm{RouteScore}}_{c_{\mathrm{cur}}}\left(t_{d,l}^{\mathrm{close}}\right)+\varepsilon_{\mathrm{sw}},$$

with $\varepsilon_{\mathrm{sw}}\in \left[0,1\right]$, after any configured minimum dwell time has elapsed. The condition acts on the third component of the reference ordering key; fresher destination sequence numbers and lower hop counts remain governed by the two higher-priority components.

An RREP received after $t_{d,l}^{\mathrm{close}}$ leaves the frozen decision unchanged. A strictly fresher late response follows native AODV freshness rules and may start a new epoch; an equal- or lower-freshness response may update permitted diagnostic or cache state and cannot reopen epoch *ℓ*. This rule bounds waiting time and makes decision timing reproducible.

Immediately before installation or switching, the adapter revalidates candidate membership, destination-sequence freshness, next-hop reachability, route lifetime, and all native AODV admissibility conditions. An infeasible provisional winner is removed and the next candidate in the frozen order is checked. Exhaustion of the set invokes native AODV discovery or repair.

Determinism, baseline-ordering equivalence on a common frozen candidate set, and candidate-admissibility preservation are proved in Section 6. The result does not assert timing equivalence between the bounded collection epoch and an implementation that installs an earlier RREP immediately.

The canonical adapter excludes compensation of additional hops by RouteScore. An experimental joint-metric adapter may introduce that trade-off after freshness filtering, provided that scaling, correctness, loop freedom, sensitivity, and performance are evaluated separately.

If an extension fits within an existing message without causing fragmentation or additional retransmissions, the number of logical control packets may remain unchanged. Nevertheless, the number of bytes, channel-occupation time, and energy consumption increase and must be measured.

The adapter selects the message format. Every RREP-extension variant accounts for the maximum transmission unit (MTU), fragmentation, retransmissions, energy, compatibility, and AODV correctness.

### 5.3. Boundary to Other Routing Families

The formal adapter, propositions, oracle, and integration trace cover AODV only for the stated reactive multi-hop WSN/MWSN profile. Other routing families are listed in Table 10 solely to delimit the claims and identify obligations for future adapters. The table is not evidence that those adapters have been implemented or validated.

Accordingly, no support is claimed for cluster-head selection, LEACH round control, RPL DODAG construction, CTP collection-tree maintenance, strictly sink-only routing, or duty-cycle-aware MAC coordination. Residual energy is not a direct RouteScore component. Each extension would require an explicit assessed object, normalized input, availability and confidence semantics, protocol-specific obligations established separately, a resource-cost model, and separate conformance and performance evaluation.

### 5.4. Behavior Under Missing Data and Failures

#### 5.4.1. Cold Start and Confidence

A neutral initialization value supports software continuity but carries zero evidential confidence. During cold start and evidence accumulation, RouteScore remains diagnostic. The adapter opens a decision gate only when the protocol-specific comparison basis is complete and confidence-sufficient. Evidence aging, timeout, module loss, or version mismatch closes the gate.

**Figure 6:**
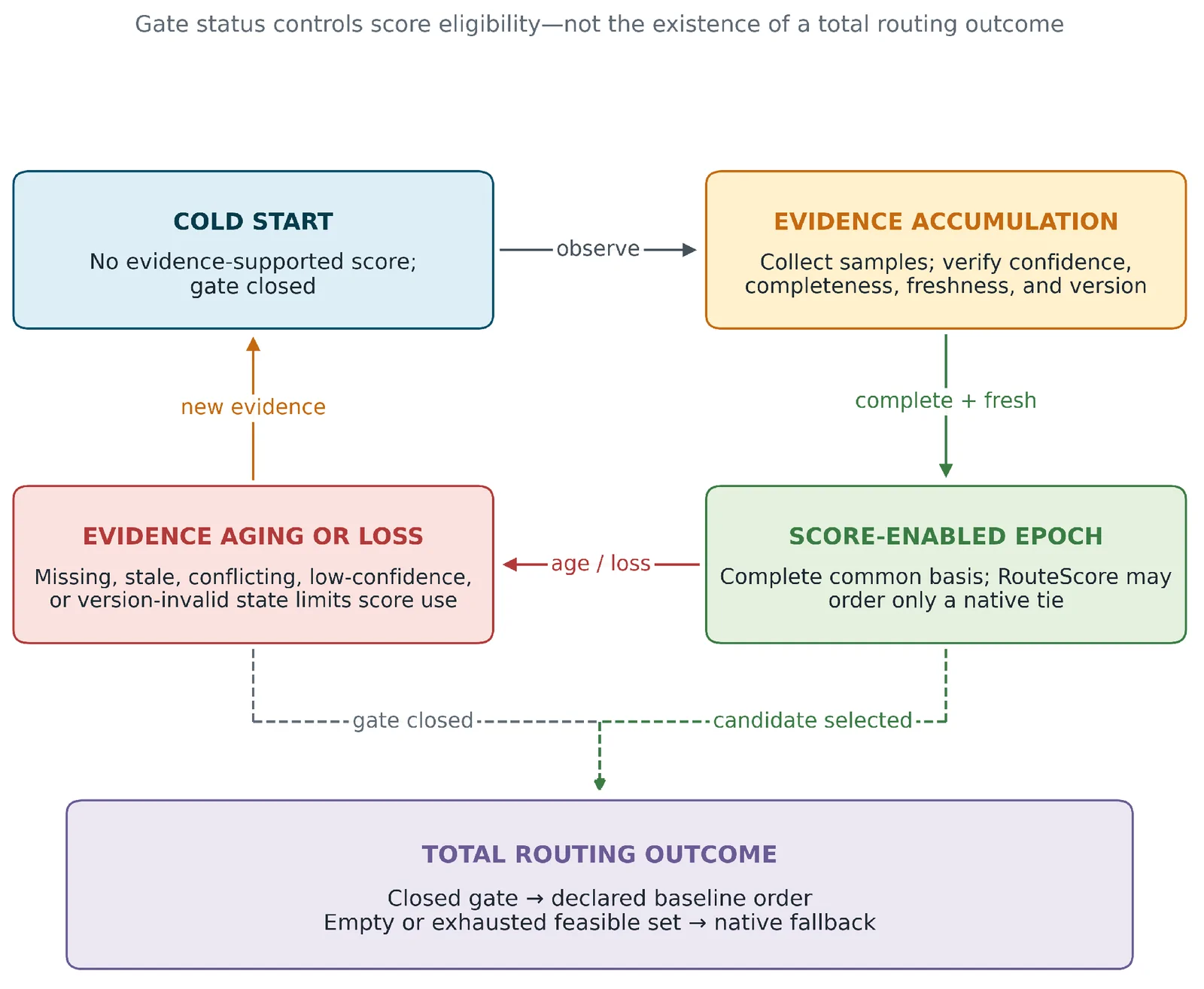
Evidence-use states of the adapter. Solid arrows show evidence-state transitions; dashed arrows show how both score-enabled and closed-gate states lead to a total routing outcome under P7.

Figure 6 summarizes this evidence-use lifecycle.

The operational gate additionally obeys the common-basis and full-coverage rules defined in this revision.

#### 5.4.2. Missing, Conflicting, and Inconclusive Evidence

Rule R-MISSING distinguishes absence from an observed adverse event. No traffic, insufficient samples, stale state, unresolved forwarding, or version mismatch removes evidence from the active basis. A confirmed negative event supplies adverse evidence. Conflicting direct and indirect reports retain provenance; reporter Trust and age limit indirect influence. Coordinated collusion remains outside the protection guaranteed by the contract.

The comparison-level rules prevent two asymmetric effects. In AODV, a missing component for one tied candidate closes the common full-basis gate. In a complete-path adapter, missing required-node Trust makes weakest-link candidate Trust unavailable. These rules preserve decision meaning, but they may increase fallback frequency when observability is poor.

The specification defines decision behavior under degraded evidence, not measured network performance. Table 11 links each condition to its formal handling and decision consequence. A closed common gate makes $A_{d,l}^{\mathrm{TA}\text{-}\mathrm{BI}}=0$, while $A_{d,l}^{\mathrm{route}}=1$ still follows under the preconditions of P7. No delivery, delay, energy, or resilience result under realistic noise is inferred from these rules.

#### 5.4.3. Topology Change, Candidate Loss, and Resource Constraints

The adapter revalidates feasibility and version immediately before commit. Candidate loss removes the stale entry from the frozen order; exhaustion invokes native discovery or repair. At low energy, an implementation may reduce update frequency, reporter count, or indirect evidence. Any such mode must expose its changed coverage and cannot silently reuse the full-evidence gate.

One-way delay from different clocks requires synchronization. Otherwise the implementation uses a single-clock quantity. Message extensions must account for bytes, airtime, fragmentation, retransmissions, and energy even when the logical packet count is unchanged.

## 6. Formal Properties and Analytical Results

The propositions below concern the mathematical state and decision contract under assumptions A1–A9. Assumption A10 bounds the WSN/MWSN application domain and resource interpretation. They establish bounded contract properties for admissible inputs: value ranges, state levels, missing-evidence neutrality, sensitivity bounds, AODV ordering on a frozen candidate set, candidate-admissibility preservation, fallback, total decisions, conditional update convergence, and a local switching guarantee. They do not establish timing equivalence, complete-protocol equivalence, or network performance.

### 6.1. Algorithmic Specification

Algorithms 1 and 2 are transition contracts over the typed records in Equation (55). Preconditions identify admissible inputs; postconditions define the state or decision that must be observable after completion.

### 6.2. Range, Level, and Missing-Evidence Properties

**Proposition 1**  (P1: Range preservation)**.** *Assume that every defined primitive input belongs to [0,1], active aggregation weights are non-negative with a positive sum, and update coefficients belong to (0,1]. Then, every defined BI, NeighborBI, RouteBI, direct Trust, Neighbor Reputation, Trust, confidence value, and RouteScore belongs to [0,1].*

**Proof.**  Active-weight normalization produces a convex combination of values in [0,1]. Exponential quality and freshness functions lie in (0,1], clipping maps every real argument to [0,1], and Equation (58) is a convex combination. Composition preserves the interval.    □

**Proposition 2**  (P2: Perturbation bound for active aggregation)**.** *Let A be a non-empty fixed active set; let $x,y\in {\left[0,1\right]}^{\left|A\right|}$, and let $\bar{w},\bar{v}$ be non-negative normalized weight vectors on A. Define the active aggregation operator by Equation (81):*

(81) $$F_{\bar{w}}\left(x\right)=C\;\left[\sum_{q\in A}{\bar{w}}_{q}x_{q}\right].$$

*Then,*

(82) $$\left|F_{\bar{w}}\left(x\right)-F_{\bar{v}}\left(y\right)\right|\leq {∥x-y∥}_{\infty }+{∥\bar{w}-\bar{v}∥}_{1}.$$

*For fixed normalized weights $\bar{w}=\bar{v}$, the aggregation is nonexpansive:*

(83) $$\left|F_{\bar{w}}\left(x\right)-F_{\bar{w}}\left(y\right)\right|\leq {∥x-y∥}_{\infty }.$$

*The fixed-weight result applies directly to BI, direct Trust, raw Trust, RouteBI, and RouteScore whenever their active set and configured normalized weights are unchanged. For NeighborBI and Neighbor Reputation, Equation (82) additionally bounds changes caused by reporter Trust, report age, freshness, or admissibility through $∥\bar{w}-\bar{v}{∥}_{1}$. The temporally stored Trust state is governed separately by the convex recursion in Equation (58) and by the conditional-convergence result in Proposition 8.*

**Proof.**  Before clipping, the difference decomposes as shown in Equation (84):

(84) $$\sum_{q}{\bar{w}}_{q}x_{q}-\sum_{q}{\bar{v}}_{q}y_{q}=\sum_{q}{\bar{w}}_{q}\left(x_{q}-y_{q}\right)+\sum_{q}\left({\bar{w}}_{q}-{\bar{v}}_{q}\right)y_{q}.$$

The first term has absolute value at most ${∥x-y∥}_{\infty }$ because $\bar{w}$ is non-negative and sums to one. Since $0\leq y_{q}\leq 1$, the second term is bounded by $∥\bar{w}-\bar{v}{∥}_{1}$. The clipping operator *C* is 1-Lipschitz, so it cannot enlarge the difference. Setting $\bar{w}=\bar{v}$ yields Equation (83).    □

**Proposition 3**  (P3: Level-consistent AODV specialization)**.** *For a fixed evaluator o, one state record per assessed node i suffices for BI, NeighborBI, and Trust, while one record per active pair (i,d) stores Route Stability, RouteBI, and RouteScore. This representation implements Equation (3) without destination-specific Trust copies.*

**Proof.**  Each pair (i,d) references the existing state keyed by *i* and adds only destination-dependent route state. Distinct destinations may therefore share ${\mathrm{Trust}}_{i}$ while retaining different RouteBI and RouteScore values. Version $\nu_{i,d}$ separates successive route instances.    □

**Proposition 4**  (P4: Common-basis missing-evidence neutrality)**.** *Under A2–A3 and A9, unavailable evidence contributes no fabricated numerical value. For a canonical complete-path candidate, missing Trust for any required forwarding node makes decision-usable candidate Trust undefined. For the reference AODV adapter, a missing positively weighted RouteScore component for any member of $C_{d,l}^{\mathrm{tie}}$ makes ${\mathrm{Comp}}_{d,l}^{\mathrm{cmp}}<1$ and therefore $\Gamma_{d,l}=0$. The complete tie class then reproduces the declared baseline order. Baseline equivalence is not asserted for optional partial-common-basis adapters.*

**Proof.**  Rule R-MISSING removes inactive data rather than inserting a scalar substitute. Equation (44) requires all required Trust states before the weakest-link minimum is decision-usable, so omission cannot raise the operational route Trust. Equations (70)–(73) require the intersection mask to contain every positively weighted component. If one is absent, the gate is zero. The third component of Equation (77) is then zero for every candidate in the tie class, leaving the total baseline rank to decide the tie.    □

**Corollary 1**  (Monotonicity of complete-basis activation)**.** *For a fixed native freshness-and-hop tie class, fixed candidate snapshots, and fixed ${\mathrm{Conf}}_{\min}$, let $\Gamma (C,Q^{+})$ denote the canonical gate for candidate set C and required positive-component set $Q^{+}$. If $C\subseteq C^{\prime }$ with $Q^{+}$ fixed, or if $Q^{+}\subseteq Q^{{+}^{\prime }}$ with unchanged weights and states for shared components and positive weights for added components, then*

(85) $$\Gamma \left(C^{\prime },Q^{{+}^{\prime }}\right)\leq \Gamma \left(C,Q^{+}\right).$$

*Thus, adding a compared candidate or a required positive component cannot open a closed gate and may close an open gate. This characterizes logical restrictiveness; it does not determine the empirical activation frequency of a mobile network.*

**Proof.**  The common mask is an intersection over candidates, so adding a candidate can only preserve or remove components. Enlarging the required positive-component set cannot improve completeness, and the confidence minimum over a larger candidate–component index set cannot increase. Each gate condition is therefore monotone non-increasing under the stated set enlargements.    □

### 6.3. Baseline Equivalence and Candidate Admissibility

**Proposition 5**  (P5: Deterministic AODV ordering and baseline equivalence)**.** *For a finite non-empty frozen RREP set satisfying the half-range assumption for sequence numbers, and for a total deterministic rank $\pi_{c}^{\mathrm{base}}$, Equation (77) selects a unique provisional candidate. RouteScore can distinguish only candidates with equal destination-sequence freshness and equal hop count. If $\Gamma_{d,l}=0$, the selected candidate equals the declared baseline winner on the same frozen set.*

**Proof.**  The comparator first applies the signed 32-bit freshness relation, then minimizes hop count. RouteScore appears only in the third component. The final component, $-\pi_{c}^{\mathrm{base}}$, resolves every remaining tie. Setting $\Gamma_{d,l}=0$ makes the RouteScore component identical throughout the tie class and reproduces the declared baseline order on the same frozen candidate set. The proposition concerns ordering, not decision latency introduced by $T_{\mathrm{RREP}}$.    □

**Proposition 6**  (P6: Candidate-admissibility preservation and native fallback)**.** *Assume that adapter A forms $C_{\mathrm{feas}}$ only from candidates accepted by the declared native routing admissibility checks, applies the declared baseline key when the gate is closed, and revalidates feasibility immediately before execution. TA-BI selects no candidate outside the commit-time feasible subset. Complete loss of usable TA-BI evidence reduces ordering to the declared native policy on the same frozen candidate set; if no candidate remains feasible, the adapter returns native fallback.*

**Proof.**  The optimization domain initially contains only candidates admitted by the declared native checks. A closed gate makes the TA-BI score component identical for the complete tie class, leaving the declared baseline key to order the same frozen set. Commit-time revalidation removes candidates that are no longer feasible. The first remaining candidate is selected; exhaustion returns fallback. These steps prove candidate-domain restriction and fallback behavior, not equivalence of timing or protocol transition traces.    □

**Scope of P6.** The proposition proves candidate-admissibility preservation, baseline ordering under a closed gate on the same frozen candidate set, and native fallback. It does not prove timing equivalence with immediate RREP installation, event-trace equivalence, liveness, route-discovery convergence, global loop freedom of the modified transition system, or preservation of every property of RFC 3561. Those broader properties require a separate protocol-level refinement analysis of the bounded RREP epoch.

**Proposition 7**  (P7: Totality of the adapter decision)**.** *For every admissible runtime state with a finite frozen candidate set and a total baseline rank, Algorithm 2 terminates and returns exactly one result: either Select(c) for a commit-time feasible candidate or $\mathrm{Fallback}(r_{f})$.*

**Proof.**  If the frozen set is empty, step 3 returns fallback. Otherwise, the signed freshness relation, hop count, optional RouteScore component, and total baseline rank induce a finite ordered list. Commit-time revalidation examines each member at most once. The first feasible member yields Select(c); exhaustion yields fallback. The cases are mutually exclusive and cover every finite input.    □

### 6.4. Convergence and Switching Stability

**Proposition 8**  (P8: Conditional convergence of a bounded update)**.** Let $X^{*}\in \left[0,1\right]$, $e_{k}=X_{k}-X^{*}$, and suppose that ${\hat{X}}_{k}=X^{*}$ for every $k\geq k_{0}$ and $\delta_{k}\geq \delta_{\min}>0$. Then,

(86) $$|e_{k}|\leq {\left(1-\delta_{\min}\right)}^{k-k_{0}}\left|e_{k_{0}}\right|⟶0.$$

**Proof.**  Subtracting $X^{*}$ from Equation (58) gives $e_{k}=\left(1-\delta_{k}\right)e_{k-1}$. Iteration and $1-\delta_{k}\leq 1-\delta_{\min}<1$ yield Equation (86).    □

**Proposition 9**  (P9: Exclusion of an immediate TA-BI-induced two-cycle)**.** *Consider two candidates a and b in the same AODV baseline tie set with an open common gate. Suppose a switch a→b at epoch k satisfies $R_{b}\left(k\right)\geq R_{a}\left(k\right)+\varepsilon_{\mathrm{sw}}$, where $R_{c}={\mathrm{RouteScore}}_{c}$, and each score changes by at most ρ before epoch k+1. If $\rho <\varepsilon_{\mathrm{sw}}$, the reverse switch b→a cannot satisfy the same hysteresis rule at epoch k+1. A positive dwell time strengthens this exclusion for every blocked epoch.*

**Proof.**  The initial switch gives $R_{a}\left(k\right)-R_{b}\left(k\right)\leq -\varepsilon_{\mathrm{sw}}$. Bounded variation gives Equation (87):

(87) $$R_{a}\left(k+1\right)-R_{b}\left(k+1\right)\leq -\varepsilon_{\mathrm{sw}}+2\rho <\varepsilon_{\mathrm{sw}}.$$

Therefore, the reverse-switch requirement $R_{a}\left(k+1\right)\geq R_{b}\left(k+1\right)+\varepsilon_{\mathrm{sw}}$ fails. The result addresses an immediate two-cycle inside one baseline tie class; higher-priority AODV changes remain authoritative.    □

### 6.5. Incremental Computational and Memory Complexity

Let $d_{i}$ be the local node degree, $m_{i}$ the number of active indirect-information entries, $q_{i}$ the number of assessed candidates, $h_{r}$ the length of route *r*, and *K* the fixed number of BI primitives. The incremental costs are

(88) $$C_{\mathrm{event}}=O\left(1\right),\;C_{\mathrm{BI}}=O\left(K\right)=O\left(1\right),\;C_{\mathrm{NeighborBI},i}=O\left(m_{i}\right),$$

(89) $$C_{\mathrm{RouteScore}}\left(r\right)=O\left(h_{r}\right),\;C_{\mathrm{cycle},i}^{\mathrm{TA}\text{-}\mathrm{BI}}=O\;\left(d_{i}+m_{i}+\sum_{r=1}^{q_{i}}h_{r}\right),$$

(90) $$M_{i}^{\mathrm{TA}\text{-}\mathrm{BI}}=O\left(d_{i}+m_{i}+q_{i}\right),\;M_{i}^{\mathrm{paths}}=O\;\left(\sum_{r=1}^{q_{i}}h_{r}\right).$$

A next-hop AODV implementation has $h_{r}=1$ at the local adapter boundary. Complete-path adapters incur route-length aggregation and storage. These bounds describe architecture-level increments; central processing unit (CPU) time, peak memory, and critical-path latency remain measured implementation outcomes.

Communication cost requires byte accounting even when TA-BI data are piggybacked. For an observation interval *T*, let $M_{i}\left(T\right)$ contain every TA-BI-originated or relayed transmission occurrence at node *i*, let $b_{m}$ be its incremental serialized byte count, and let $I_{m}^{\mathrm{standalone}}$ indicate a separate logical packet. Then,

(91) $$\Delta B_{i}\left(T\right)=\sum_{m\in M_{i}\left(T\right)}b_{m},\;\Delta N_{i}\left(T\right)=\sum_{m\in M_{i}\left(T\right)}I_{m}^{\mathrm{standalone}},$$

(92) $$\Delta B_{\mathrm{network}}\left(T\right)=\sum_{m\in M(T)}h_{m}b_{m},$$

Here, M(T) contains distinct source reports and $h_{m}$ is the number of transmitting hops for report *m*. Passive local observation adds no TA-BI report transmission. Piggybacking can yield $\Delta N_{i}\left(T\right)=0$ while $\Delta B_{i}\left(T\right)>0$; logical packet count alone is therefore insufficient. Table 12 separates analytical bounds from quantities that require a target implementation and radio model.

### 6.6. Claim–Proof–Test Traceability

Table 13 gives each formal claim a unique identifier and links it to its assumptions, proof, and executable evidence. The oracle tests are supporting examples of boundary behavior; the mathematical proofs remain the basis of the propositions.

## 7. Executable Conformance and Bounded Integration Reachability

The evidence is organized by inference level. Proofs support formal correctness; the deterministic oracle and fixed profiles support executable conformance; the bounded historical OMNeT++/INET record supports integration reachability only; and empirical network performance is not evaluated. Evidence at one level is not used to infer a claim at a higher level.

**Figure 7:**
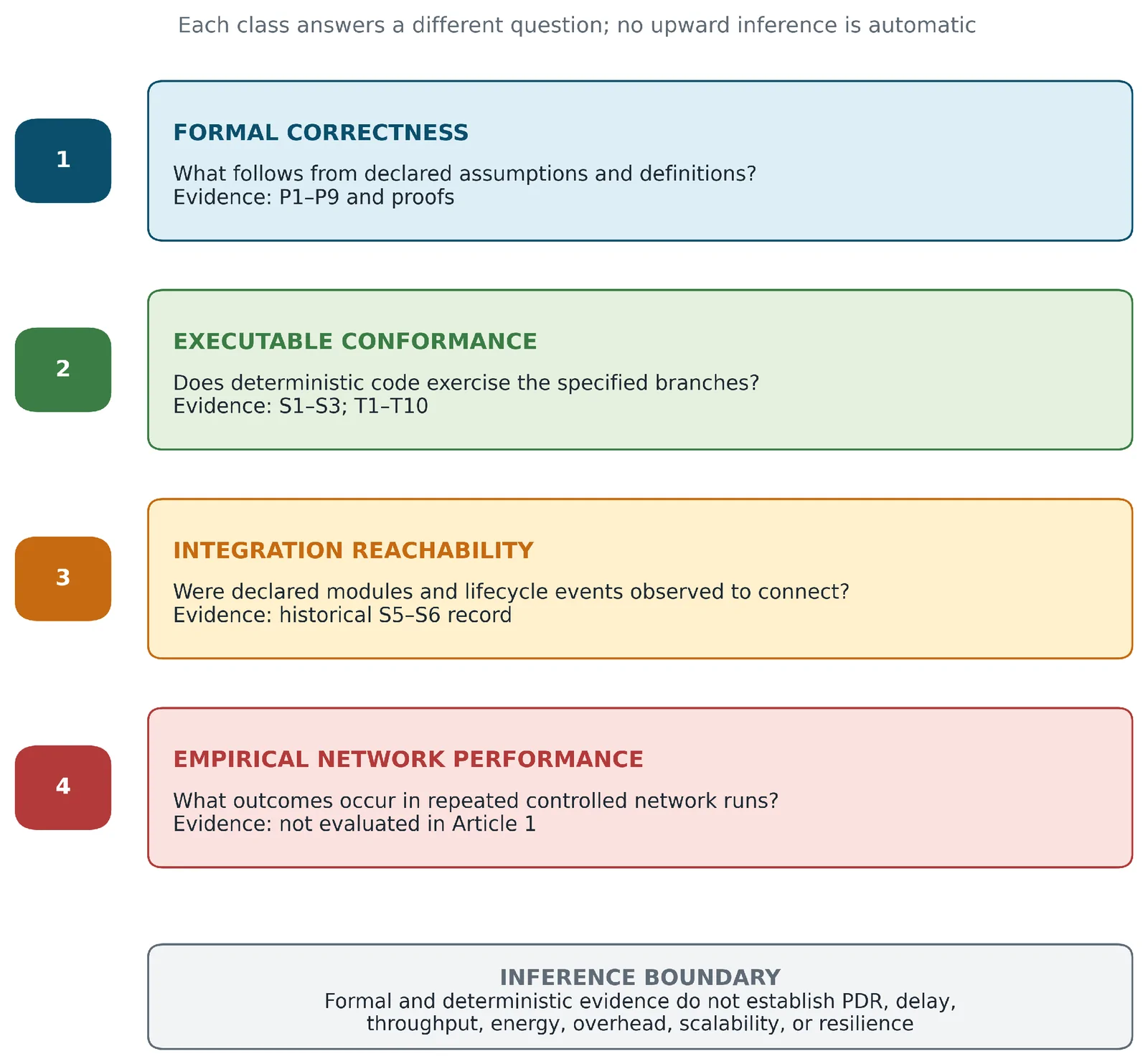
Four-level evidence taxonomy and inference boundary. Each class answers a different question; evidence in one class does not establish a claim in a higher empirical class.

Figure 7 summarizes the evidence chain.

### 7.1. Reference Oracle and Ten Conformance Groups

Supplementary File S1 implements active aggregation, full-path Trust completeness, the AODV common component mask, signed sequence freshness, final revalidation, fallback, bounded updates, hysteresis, and version checks. T3 now contains subcases T3a–T3c for state keys, frozen-version rejection, expiry, and closed-epoch isolation. T4 contains subcases T4a–T4d for asymmetric missing evidence, low confidence, incomplete path Trust, and restoration of a complete fresh basis in a later epoch. Supplementary File S2 contains the deterministic output. Table 14 maps the ten test groups to formal claims.

Files S1–S3 constitute the rerunnable evidence set. S1 was validated with CPython 3.13.5; it uses the standard library only, invokes no stochastic operation, and therefore requires no random seed. From the supplementary directory, run python3 TA-BI_conformance_oracle.py. The command reproduces the S2 line SUMMARY 10/10 test groups passed only when every T3/T4 subcase and every other group passes. S3 contains the complete fixed input/output records for the five decision-semantic profiles. File S7 records the command, software boundary, and SHA-256 checksums of the final package.

### 7.2. Fixed AODV–TA-BI Decision-Semantic Profiles

Five fixed deterministic profiles provide decision-semantic witnesses for the conceptual and formal stage. They compare the native AODV order with the reference TA-BI adapter on the same frozen candidate set; they are not a network-performance experiment and do not simulate delivery, delay, radio loss, traffic, or energy. The complete machine-readable input and output are provided as Supplementary File S3. Table 15 summarizes the five decision profiles and the interpretation supported by each outcome.

### 7.3. Historical OMNeT++/INET Integration-Reachability Record

A bounded historical proof of concept used OMNeT++ 6.3.0 and INET 4.5.4. Its retained summary records the module path AODV → analyzer → behavioral and Trust processing → state tables → decision engine → AODV and a route-lifecycle event sequence. These observations support integration reachability only. Numerical packet counts and event times remain historical metadata in S5 and are not used as main-text evidence.

The exact raw log, scalar/vector files, seed, and complete source snapshot for that historical run are not contained in the supplied manuscript archive. Supplementary File S5 records the bounded integration-trace summary, whereas Supplementary File S6 records the artifact-availability boundary. Because the exact bundle is unavailable, the trace is excluded from the rerunnable evidence set and supports no formal-correctness, executable-conformance, or network-performance inference.

### 7.4. Evidence Boundary

Table 16 states the four-level taxonomy used throughout the article. The decision-semantic chain is definition → proposition → oracle assertion → deterministic profile. The simulator record is a separate module-to-event trace rather than another link in that proof chain.

## 8. Potential Applications

TA-BI targets sensor-oriented deployments in which a reactive routing process exposes multiple feasible candidates and the evidence can be assigned to a node, link, or route instance. The following application classes motivate separate validation strata rather than universal performance claims:

- **Environmental WSNs:** Long-lived monitoring with intermittent interference, lossy links, and strict energy budgets;
- **Industrial WSNs:** Bounded latency, predictable fallback, controlled reporting overhead, and traceable security decisions;
- **Mobile sensing:** Sensing nodes attached to people, robots, assets, or vehicles, with changing neighborhoods and evidence age;
- **Emergency MWSNs:** Mobile sensors, relays, or sinks operating under damage, partitions, rapid topology change, and incomplete observations;
- **Mobile relay-assisted WSNs:** Planned or opportunistic relay motion used to restore collection paths or extend coverage.

Each application requires its own radio, traffic, energy, mobility, security, and timing profile. General-purpose MANET systems remain outside the application claims.

## 9. Discussion

### 9.1. Interpretation of the Contribution

The contribution is an integration contract for reactive multi-hop WSNs and MWSNs. TA-BI gives current observations, historical node Trust, candidate state, and native routing information separate roles. It then connects them through an adapter with explicit missing-evidence, fallback, and version rules. The narrowed scope strengthens the interpretation: the architecture is designed for constrained sensor-oriented nodes and collection endpoints, not for every form of mobile peer-to-peer networking.

The state separation is important in MWSNs because mobility can simultaneously change link quality, evidence availability, and candidate identity. A short-lived degradation should not automatically rewrite long-term Trust. Conversely, historical Trust should not conceal a currently unusable route. Typed keys, bounded update rules, and route-instance versions make these distinctions executable.

The common-basis revision resolves a substantive weakness in candidate comparison. Candidate-local renormalization is appropriate for diagnostic values from partial evidence, but it is not sufficient for fair ranking. The revised AODV adapter intersects the masks of all candidates in one native tie class and requires the complete configured basis before opening the gate. The rule may reduce the number of TA-BI decisions, but every open-gate comparison has a consistent meaning.

The complete-path rule addresses the same problem at node-aggregation level. A weakest-link minimum over only available nodes can increase when a poorly performing relay becomes unobservable. Full required-node coverage prevents that increase from becoming operational. Partial values remain useful for diagnostics or observation scheduling, but they are not treated as decision-usable route Trust.

### 9.2. Relation to Established and Recent Sensor-Network Routing

TARF and LDTS demonstrated that trust can guide WSN routing while controlling resource use [5,6]. Recent WSN and underwater-WSN methods combine trust with clustering, energy, topology, fuzzy logic, or learned policies [16,17,18,19,20]. MWSN studies additionally emphasize mobility roles, collection continuity, and repair under changing connectivity [1,2]. These works motivate the TA-BI evidence space, but they do not define the same integration objective.

TA-BI does not claim to replace or outperform those estimators and routing schemes. It specifies how a current-quality estimator, a historical Trust process, and a candidate metric must be typed, gated, versioned, and connected to a protocol decision. An estimator can be substituted only after its outputs, confidence, completeness, resource cost, and native protocol obligations are mapped explicitly.

### 9.3. Why AODV Is a Suitable Reference Adapter for Reactive MWSNs

AODV is not presented as the universal routing solution for WSNs or MWSNs. It is selected as a reference adapter because its on-demand discovery exposes RREQ/RREP events, explicit next-hop–destination candidates, destination-sequence freshness, and hop-count precedence. These elements create a clear boundary at which an auxiliary RouteScore can be introduced without redefining the entire routing protocol. AODV-derived approaches have also been studied in sensor-network settings where energy and sink-oriented delivery remain important [4].

This choice is strongest for reactive multi-hop MWSNs with intermittent communication demands, changing connectivity, and more than one feasible response to route discovery. It is weaker for deployments organized primarily as stable collection trees, DODAGs, or rotating clusters. RPL, CTP, and LEACH use different assessed objects and correctness constraints. They therefore require separate adapters rather than lexical replacement of protocol names.

The bounded RREP collection epoch is also a conscious design trade-off. Waiting can expose a reproducible tie class and allow common-basis comparison. It can increase setup delay and consume additional receive time on constrained nodes. The reference profile therefore establishes semantic correctness, not energy optimality. A WSN/MWSN performance study must measure discovery latency, radio-on time, control bytes, and energy alongside delivery outcomes.

### 9.4. What TA-BI Does Not Replace

The architecture does not replace authentication, integrity, replay protection, identity management, or attack-specific detection. Provenance metadata can carry the result of those mechanisms, but it cannot create authenticated identity. Likewise, RouteScore does not establish feasibility. The routing protocol still performs discovery, validity checks, sequence handling, loop prevention, repair, and final commitment.

TA-BI also does not replace cluster-head election, DODAG construction, collection-tree maintenance, data aggregation policy, sink scheduling, or duty-cycle-aware MAC control. The reference RouteScore does not directly optimize residual energy. Energy may influence a future sensor-network profile only through an explicit component and only after its effect on fairness, lifetime, and the protocol-specific adapter obligations has been defined.

Observable behavior is not equivalent to malicious intent. Collision, hidden terminals, mobility, congestion, asymmetry, sleep schedules, and energy depletion can resemble non-cooperative forwarding. TA-BI distinguishes unresolved absence from confirmed adverse evidence. This reduces false attribution at the contract level, but classification accuracy remains dependent on instrumentation and scenario design.

### 9.5. Decision Timing and the Bounded RREP Epoch

The bounded RREP epoch creates a latency and resource trade-off. Waiting allows several native-feasible candidates to be compared on one frozen set. It can improve decision reproducibility and enable a meaningful tie class. It can also delay route installation and keep a constrained radio active longer than an implementation that accepts the first adequate RREP. The formal baseline-equivalence result concerns ordering on the same frozen set, not end-to-end decision latency or energy.

A deployment must report $T_{\mathrm{RREP}}$, discovery deadlines, response arrival distributions, setup delay, control traffic, and radio-on time. An adaptive epoch would require a separate stopping rule and proof that late evidence cannot change a committed comparison inconsistently.

### 9.6. Partial Observability, Collusion, and Selection Bias

Common-basis gating prevents asymmetric missing components from directly favoring one candidate, but it does not eliminate partial-observability bias. Frequently used routes generate more forwarding evidence than inactive routes. In an MWSN, mobility can amplify this imbalance by repeatedly moving some relays into and out of observation range. The system may become more certain about the current route and repeatedly fall back for alternatives. Optional exploration can reduce this self-confirming pattern, but exploration consumes energy, traffic budget, and risk. It must remain a separate policy with explicit limits.

Indirect evidence increases coverage but creates exposure to collusion and reputation poisoning. Reporter Trust, freshness, provenance, and bounded influence reduce individual impact. They do not guarantee resistance to coordinated identities or correlated false reports. Authentication and diversity constraints are external requirements. Ablation should compare local-only, indirect-enabled, and adversarial-report variants before operational claims are made.

### 9.7. Practical Consequences of Completeness

The complete common-basis gate prioritizes comparability over the frequency of score-enabled decisions. Under P7, a routing outcome remains total because a closed gate invokes baseline ordering or native fallback; only TA-BI enhancement availability is reduced. The ratio ${\hat{\rho }}^{\Gamma }$ is defined for empirical reporting but is not estimated here. A low gate-activation rate is not necessarily undesirable: it may reveal sparse contacts, mobility-driven observation gaps, or strict thresholds. Conversely, a high rate may indicate permissive thresholds or that initialization values are being mistaken for evidence. Future experiments should report gate activation, closed-gate reasons, common-mask composition, path Trust coverage, evidence age, version rejection counts, and mobility state alongside packet-level metrics.

The architecture-level cost bounds remain useful for WSN/MWSN implementation planning. Local observation adds no report traffic. Indirect evidence adds serialized bytes, per-hop transmissions, processing, storage, and radio-on time. These costs depend on reporter count, cache lifetime, update interval, mobility, and duty cycle. They must be measured on the same hardware and radio model used for performance claims.

### 9.8. Formal Correctness, Executable Conformance, Integration Reachability, and Network Performance

Formal correctness means that a proposition follows from declared assumptions and definitions. Executable conformance means that deterministic code exercises specified state, ordering, availability, or fallback rules. Integration reachability means only that declared modules and route-lifecycle events were observed to connect in a bounded simulator trace. Empirical network performance means measured end-to-end outcomes from repeated controlled runs with uncertainty reporting. The proofs support the first level, S1–S3 support the second, and the historical S5 and S6 record supports only the third. The fourth level is not evaluated.

The next project stage should use paired AODV and TA-BI runs with identical WSN/MWSN topology, mobility roles, traffic, attack schedule, seeds, and build state. It should report delivery ratio, delay distribution, route setup and recovery time, route switches, control bytes, indirect-report bytes, radio-on time, energy, CPU time, memory, and uncertainty intervals. Parameter sensitivity and ablation are necessary because the common-basis rule, confidence threshold, $T_{\mathrm{RREP}}$, and hysteresis margin can trade decision availability for stability and energy expenditure.

### 9.9. Threats to Validity

The literature review is selective rather than systematic. Matrix codes are constrained by the operational definitions, domain labels, and source locators in S4, but another coding scheme may yield different classifications. The formal propositions depend on A1–A9; A10 bounds the WSN/MWSN deployment claims and cost interpretation. The propositions do not establish global closed-loop stability, energy efficiency, or mobility robustness. The hysteresis result excludes only an immediate TA-BI-induced reverse switch inside one native tie class.

The AODV specialization represents one reactive sensor-network profile. It does not establish suitability for all WSN/MWSN routing families or for general-purpose MANETs. The OMNeT++ trace is historical integration evidence; the exact raw run bundle is not included in the current archive. These boundaries are disclosed so that no performance, domain-generalization, or reproducibility inference exceeds the supplied evidence.

## 10. Conclusions

Within the stated reactive multi-hop WSN/MWSN application scope, TA-BI provides a coherent observation-to-decision contract for trust-aware routing support. Current BI and NeighborBI remain distinct from historical node Trust. Candidate RouteBI and RouteScore retain destination or path context. The native routing protocol remains responsible for feasibility and final commitment.

The architecture addresses sensor-network conditions in which constrained sensing, relay, or gateway nodes may be static or mobile and may expose several feasible route candidates toward a sink, gateway, or data-collection endpoint. MANET research remains relevant as the origin of AODV, behavioral monitoring, reputation, and formal protocol analyses, but the article does not claim validation for the general MANET domain.

The complete common-basis gate closes two missing-evidence gaps. AODV candidates use RouteScore only on one complete shared mask; otherwise the declared baseline tie policy is reproduced. Complete-path weakest-link Trust requires full required-node coverage. Routing-decision availability remains distinct from TA-BI enhancement availability, and ${\hat{\rho }}^{\Gamma }$ provides a future reporting quantity for the latter. The monotonicity corollary states the logical restrictiveness of the complete basis without estimating its frequency in MWSNs.

Nine propositions support formal correctness, and ten oracle groups—including expanded T3/T4 subcases—support executable conformance to the tested branches. A five-profile comparison shows where TA-BI may change ordering inside an AODV freshness-and-hop tie and where native precedence, missing evidence, or revalidation remain authoritative. The historical OMNeT++/INET record supports integration reachability only.

For the reference adapter, P6 is limited to candidate-admissibility preservation, native precedence in the declared ordering key, baseline ordering on the same frozen set when the gate is closed, commit-time revalidation, and native fallback. Because the bounded RREP epoch changes collection and commitment timing, no timing or event-trace equivalence, liveness, global loop freedom of the modified transition system, or preservation of every RFC 3561 property is claimed. Packet delivery, delay, throughput, energy, overhead, scalability, mobility, and resilience remain empirical network-performance questions.

## Figures and Tables

**Table 1:** Evidence-semantic coding of representative trust-aware and verification approaches. Codes: Y, explicit; P, partial; N, absent; N/A, not applicable. Domain codes and source locators are provided in Supplementary File S4.

Approach and Primary Domain	Current/ Historical	Node/ Candidate	Provenance	Missing Evidence	Confidence
Watchdog/Pathrater (MANET) [22]	P	P	P	N	N
CORE/CONFIDANT (MANET) [23,24]	P	P	P	N	P
TARF (WSN) [5]	P	P	P	N	P
LDTS (WSN) [6]	P	P	P	N	P
TAODV/AOTDV (MANET) [25,26]	N	P	P	N	P
T2AR (MANET) [27]	P	P	P	N	P
Void-avoiding trust routing (UWSN) [16]	P	P	P	P	P
GTR (UWSN) [17]	P	P	P	P	P
ACTAR (WSN/IoT) [18]	P	P	P	P	P
Fuzzy multipath (WSN) [19]	P	P	P	P	P
Context-aware trust prediction (opportunistic IoT) [21]	P	P	P	P	P
TEAHR (WSN) [20]	P	P	P	P	P
Formal AODV analyses (AODV/MANET) [29,30,31]	N/A	N/A	N/A	N/A	N/A
TA-BI (target: WSN/MWSN)	Y	Y	Y	Y	Y

**Table 2:** Routing-integration, fallback, verification, implementation, and cost coding. Codes: Y, explicit; P, partial; N, absent; N/A, not applicable. Operational definitions and source locators are provided in Supplementary File S4.

Approach and Primary Domain	Native Admission	Fallback	Sequence Number	Formal Properties	Executable Support	Cost Model
Watchdog/Pathrater (MANET) [22]	P	P	N/A	N	Y	P
TARF/LDTS (WSN) [5,6]	P	P	N/A	N	Y	P
TAODV/AOTDV (MANET) [25,26]	P	P	P	N	Y	P
T2AR (MANET) [27]	P	P	P	N	Y	P
Void-avoiding trust routing (UWSN) [16]	P	P	N/A	N	Y	P
GTR (UWSN) [17]	P	P	N/A	N	Y	P
ACTAR (WSN/IoT) [18]	P	P	N/A	N	Y	P
Fuzzy multipath (WSN) [19]	P	P	N/A	N	Y	P
Context-aware trust prediction (opportunistic IoT) [21]	P	P	N/A	N	Y	P
TEAHR (WSN) [20]	P	P	N/A	N	Y	P
Formal AODV analyses (AODV/MANET) [29,30,31]	Y	N/A	Y	Y	Y	N/A
TA-BI (target: WSN/MWSN)	Y	Y	Y	Y	Y	Y

**Table 3:** Object types used by the analytical core and protocol adapters.

Symbol	Object Type	Formal Role	Reference AODV Example
*o*	Evaluator node	Local context that owns observations and state	Node executing TA-BI
*i*	Assessed node	Node-level BI, NeighborBI, FR, LS, NR, and Trust key	Candidate next hop
*d*	Destination	Native routing context	Destination IP address
*c*	Generic candidate	Adapter-visible object eligible for ranking	RREP-derived candidate $c_{\left(i,d\right)}$
*r*	Complete route	Candidate exposed as an ordered path	Source route in a complete-path protocol
$\nu_{i,d}$	Route-instance version	Version of candidate state, not candidate identity	Version of the current next-hop–destination association

**Table 4:** Core identifiers and operators in the TA-BI formal model.

Symbol	Meaning	Domain/Level	Source and Availability
*o*	Fixed local evaluator; suppressed in compact notation	Identifier/node	Local TA-BI execution context exists
*i*	Assessed node; next hop in the AODV specialization	Identifier/node	Active or observable neighbor state
*j*	Observer or reporter	Identifier/node	Authenticated and identifiable direct or indirect source
*d*	Routing destination	Address or identifier/route context	Valid destination in native routing state
*c*	Generic candidate admitted by the protocol adapter	$c\in C_{\mathrm{feas}}\left(t\right)$/adapter	Declared adapter admissibility conditions satisfied
*r*	Complete route or its protocol representation	Sequence or ID/route	Current route instance is available
$\nu_{i,d}$	Version of local entry (i,d)	Tag/adapter	Created from installation time, sequence state, or canonical activation event
W(t)	Local observation window	Time interval/observation	Local clock; Δt>0
C(x)	Clipping operator	[0,1]/analytical core	Defined for every real input
$V\left(r\right),h_{r},v_{k}$	Forwarding nodes of route *r*, route length, and element *k*	Set and integer/route	Complete-path adapter exposes the route representation
$V_{\mathrm{valid}}^{X}$	Elements with current, sufficiently confident state *X*	Subset of *V*/route	Non-empty after availability, age, and confidence filtering
$\delta_{X,i,k}$	Update coefficient of state *X* at step *k*	(0,1]/core	Explicitly configured; δ=1 is memoryless

**Table 5:** Node- and route-level TA-BI states and their evidence-availability conditions.

Symbol	Meaning	Domain/Level	Evidence and Availability Condition
${\mathrm{BI}}_{i}$	Current local communication quality associated with *i*	[0,1] or N/A/node	At least one current positively weighted loss or delay component
${\mathrm{NeighborBI}}_{i}$	Behavioral Information reported about *i*	[0,1] or N/A/node	Positive admissible reporter-weight sum with sufficient freshness and confidence
$S_{r},S_{i,d}$	Stability of a full route or local AODV route instance	[0,1]/route	Valid route instance and canonical route-change record
${\mathrm{RouteBI}}_{i,d}$	Behavioral assessment of AODV candidate (i,d)	[0,1] or N/A/candidate	At least one positively weighted current component among BI, NeighborBI, and Route Stability
${\mathrm{FR}}_{i}$	Execution ratio of expected forwarding	[0,1] or N/A/node	$N_{\mathrm{expected},i}>0$ and observations are resolvable
${\mathrm{LS}}_{i}$	Stability of the local neighbor link	[0,1] or N/A/node or link	Local link is observable
${\mathrm{NR}}_{i}$	Neighbor Reputation	[0,1] or N/A/node	Positive admissible reporter-weight sum
${\mathrm{Trust}}_{i}$	Accumulated operational reliability of node *i*	[0,1] or N/A/node	At least one current active component or a still-current evidence-supported stored state

**Table 6:** Candidate-level scores, confidence metadata, and their availability conditions.

Symbol	Meaning	Domain/Level	Evidence and Availability Condition
${\mathrm{Trust}}_{c}$	Candidate-level aggregation of node Trust	[0,1] or N/A/adapter	$V_{\mathrm{valid}}^{\mathrm{Tr}}\left(c,t\right)$ is non-empty
${\mathrm{RouteScore}}_{i,d}$	Reference AODV candidate score	[0,1] or N/A/candidate	At least one positively weighted active component; the all-components AODV equation applies when both RouteBI and Trust are available
${\mathrm{RouteScore}}_{c}$	Generic candidate score	[0,1] or N/A/adapter	Candidate is feasible and its active RouteScore weight sum is positive
${\mathrm{Conf}}_{c}^{q}$	Confidence of RouteScore component *q*	[0,1]/adapter	Computed from $N_{c,q}^{\mathrm{obs}}$, $N_{\min,q}$, age, and resolvability
${\mathrm{Conf}}_{c}$	Confidence of the candidate evidence basis	[0,1]/adapter	Non-empty active RouteScore set; otherwise zero
$g_{c}$	Gate controlling RouteScore use	{0,1}/adapter	Always computable from ${\mathrm{Conf}}_{c}$ and ${\mathrm{Conf}}_{\min}$

**Table 7:** State semantics by level, time horizon, primary source, and historical-update rule.

State	Level	Time Horizon	Primary Source	Historical Update
${\mathrm{BI}}_{i}$	Node	Current observation window	Local loss and delay evidence concerning *i*	No; recomputed from the current active set
${\mathrm{NeighborBI}}_{i}$	Node	Current report horizon	Admissible reports about current communication quality	No; recomputed after freshness and provenance filtering
${\mathrm{Trust}}_{j\to i}^{\mathrm{direct}}$	Observer–node pair	Current observation window	Forwarding Ratio and Link Stability observed by *j*	No; direct current assessment
${\mathrm{NR}}_{i}$	Node	Current report horizon	Weighted direct assessments supplied by admissible reporters	No; current indirect aggregate
${\hat{\mathrm{Trust}}}_{i}$	Node	Current update step	Active combination of FR, LS, and NR	No; raw input to the stored state
${\mathrm{Trust}}_{i}$	Node	Multiple update steps	Previous stored Trust and defined raw Trust	Yes; Equation (58)
${\mathrm{RouteBI}}_{c}$	Candidate	Current candidate instance	BI, NeighborBI, and Route Stability mapped by the adapter	No; recomputed for version $\nu_{c}$
${\mathrm{RouteScore}}_{c}$	Candidate	Current decision epoch	Active RouteBI and candidate Trust components	No in the reference baseline; filtering requires a separate declared variant

**Table 8:** Adapter, missing-evidence, and discrete-update symbols used by the reference specification.

Symbol	Definition, Domain, and Operational Role
$a_{i}^{X}\left(t\right)$	Availability indicator, $a_{i}^{X}\left(t\right)\in \left\{0,1\right\}$; equal to one only for current, resolvable, evidence-supported data concerning component or state *X*.
$\rho_{j,i}\left(t\right)$	Reporter weight in Neighbor Reputation, $\rho_{j,i}\left(t\right)\in \left[0,1\right]$; combines prior reporter Trust, freshness, and provenance admissibility.
$A_{X,i,k}$	Normalized observation variability, $A_{X,i,k}\in \left[0,1\right]$; optional input to the adaptive update rule.
$Q_{i,k}$	Current-data reliability, $Q_{i,k}\in \left[0,1\right]$; combines sample coverage and information age.
$U_{c}^{\mathrm{base}}\left(t\right)$	Native protocol information for candidate *c*; a protocol-specific input to the generic adapter mapping $\Phi_{A}$.
$\Phi_{A},\;\Phi_{\mathrm{AODV}}$	Generic and AODV-specific mappings from native information and TA-BI metadata to an ordering key; each must reproduce the declared baseline ordering when TA-BI influence is disabled.
${≻}_{A}$, $K_{c}^{A}$	Adapter ordering relation and the resulting scalar or tuple key actually used for candidate comparison.
$\pi_{c}^{\mathrm{base}}\in N$	Deterministic baseline tie rank; a lower value is preferred. The reference AODV policy derives it from accepted-RREP event order and next-hop address.
$\Gamma_{d,l}\in \left\{0,1\right\}$	Common gate for the baseline-equivalent AODV tie set in epoch *ℓ*; one only when every candidate in that set has an open gate.
$T_{\mathrm{RREP}},t_{d,l}^{\mathrm{open}},t_{d,l}^{\mathrm{close}}$	RREP collection duration and decision-window boundaries, with $t_{d,l}^{\mathrm{close}}=t_{d,l}^{\mathrm{open}}+T_{\mathrm{RREP}}$.
$c_{\mathrm{base}},c_{\mathrm{cur}},c_{\mathrm{new}}$	Baseline winner, currently installed candidate, and challenger used by fallback and switching logic.
$\varepsilon_{\mathrm{sw}}\in \left[0,1\right]$	Minimum RouteScore improvement required for a TA-BI-induced switch within an AODV tie set; applied to the third component of the reference ordering key.

**Table 9:** Parameter roles, admissible domains, and selection obligations.

Parameter Group	Formal Domain	Role	Selection and Reporting Rule
Aggregation weights	Non-negative; positive active sum	Combine normalized components	Declare all raw and normalized weights; justify by domain requirements or calibration data
Update coefficients, $\delta_{X}$	$0<\delta_{X,\min}\leq \delta_{X}\leq \delta_{X,\max}\leq 1$	Historical memory and response rate	Relate to observation interval and desired memory horizon; report bounds and nominal values
Age and confidence controls	Positive age constants; ${\mathrm{Conf}}_{\min}\in \left[0,1\right]$; $N_{\min,q}>0$	Evidence usability and gate state	Derive from sampling and instrumentation reliability; report threshold-crossing frequency
$T_{\mathrm{RREP}}$	Positive and no longer than native discovery deadline	Frozen candidate collection	Relate to RREP-arrival distribution and deadline; report setup delay and late responses
$\varepsilon_{\mathrm{sw}}$ and dwell time	$\varepsilon_{\mathrm{sw}}\in \left[0,1\right]$; dwell time non-negative	Local switching resistance	Relate to score variability and application switching cost; report switch counts

**Table 10:** AODV reference scope and adapter obligations for other routing families.

Protocol or Family	Status in This Article	Adapter Obligation Outside This Article
AODV [3]	In-scope reference adapter for reactive multi-hop WSNs/MWSNs	Preserve sequence-number freshness, route validity, hop-count precedence, next-hop reachability, route lifetime, and final revalidation
RPL [32]	Out of scope; no DODAG adapter is claimed	Define the parent-candidate object and Objective Function contribution while preserving Rank monotonicity, DODAG consistency, and loop freedom
CTP [33]	Out of scope; no collection-tree adapter is claimed	Define how evidence enters parent selection while preserving collection-tree consistency, datapath validation, and adaptive control behavior
LEACH [34]	Out of scope; no cluster-head adapter is claimed	Define a cluster-head candidate, round boundary, rotation rule, and energy criterion before any Trust contribution
Geographic routing (GPSR) [35]	Out of scope; no geographic adapter is claimed	Restrict assessment to candidates admitted by greedy progress or the active recovery procedure
DSR/OLSR [36,37]	MANET protocol lineage only	Define source-route or MPR candidate semantics and preserve the corresponding native validity and coverage rules

*Abbreviations:* AODV, Ad Hoc On-Demand Distance Vector; RPL, IPv6 Routing Protocol for Low-Power and Lossy Networks; DODAG, destination-oriented directed acyclic graph; CTP, Collection Tree Protocol; LEACH, Low-Energy Adaptive Clustering Hierarchy; GPSR, Greedy Perimeter Stateless Routing; DSR, Dynamic Source Routing; OLSR, Optimized Link State Routing; MPR, multipoint relay.

**Table 11:** Specified decision behavior under degraded evidence and its evidential support.

Condition	Formal Handling	Decision Consequence	Evidence
Missing	Availability zero; omit from numerator and denominator	Close the common AODV gate if the complete basis is lost; use baseline ordering	P4; T1, T4a
Stale/version mismatch	Fail age or version check; stored scalar remains unavailable	Exclude from the decision basis; reject stale candidate state; do not reopen a frozen epoch	A3, A8; T3b–T3c
Conflicting	Retain provenance; bound admissible indirect influence by reporter Trust and age	May reduce confidence or close the gate; coordinated collusion is not resolved by the contract	R-MISSING; Section 5.4
Noisy	Clip values; apply P2 bounds, bounded historical update, and hysteresis	Small changes remain bounded for a fixed active set; threshold crossing may change the active set	P1, P2, P8, P9; T2, T9, T10
Low confidence	Keep diagnostic values but fail the common-gate threshold	RouteScore is ineligible; baseline ordering remains available	P4, P5; T4b
Restored basis	Re-evaluate only in a later version-consistent epoch	A complete fresh basis can reopen the gate for the later epoch	T4d

**Table 12:** Incremental resource accounting and evidence status.

Resource	Quantity or Bound	Evidence Status
Processing	$C_{\mathrm{event}}=O\left(1\right)$; $C_{\mathrm{BI}}=O\left(1\right)$; $C_{\mathrm{NeighborBI},i}=O\left(m_{i}\right)$; cycle bound in Equation (89)	Architecture-level bound
Storage	Equation (90); additional complete-path storage $O(\sum_{r}h_{r})$	Architecture-level bound
Communication	$\Delta N_{i}\left(T\right)$, $\Delta B_{i}\left(T\right)$, and per-hop $\Delta B_{\mathrm{network}}\left(T\right)$	Exact accounting identities; values require an implementation trace
CPU, latency, radio, energy	CPU time, peak bytes, critical-path delay, airtime, retransmissions, radio-on time, joules	Empirical measurements; not claimed here

**Table 13:** Traceability from formal claims to assumptions, proofs, and executable-conformance tests.

ID	Assumptions	Claim	Proof	Executable Evidence
P1	A1–A3	Every defined BI, NeighborBI, Trust, RouteBI, and RouteScore value remains in [0,1]	Proposition 1	T1 aggregation range
P2	A1–A3	Active aggregation is nonexpansive for fixed normalized weights and bounded for changing weights	Proposition 2	T2 perturbation assertions
P3	A3, A8	Node state and candidate state use different keys and candidate versions cannot be mixed	Proposition 3	T3 key and version checks
P4	A2–A3, A9	Complete-path Trust requires full node coverage; AODV candidates share one complete component mask or use baseline order	Proposition 4	T1 and T4
P5	A4–A5, A8–A9	RouteScore acts only after sequence freshness and hop count; a closed gate reproduces baseline order	Proposition 5	T4–T6
P6	A5, A8–A9	Only commit-time feasible candidates can be selected on the frozen set; exhausted candidates lead to native fallback	Proposition 6	T7
P7	A4–A5, A8	Every finite admissible decision returns exactly one select or fallback result	Proposition 7	T8
P8	A1, A6	A bounded update converges geometrically to a stationary target under persistent observations	Proposition 8	T9 update recursion
P9	A7	A RouteScore change smaller than the hysteresis margin cannot cause an immediate reverse switch	Proposition 9	T10 hysteresis sequence

**Table 14:** Executable-conformance groups and the formal claims they exercise.

ID	Boundary Condition	Required Result	Formal Claim
T1	Active aggregation with unavailable input	Defined output remains in [0,1]; absent input has zero numerical contribution	P1, P4
T2	Fixed and changed normalized weights	Differences satisfy Equations (82) and (83)	P2
T3	State keys, stale version, expired evidence, and late update	Node/candidate levels remain distinct; stale or expired state is rejected and cannot reopen a frozen epoch	P3
T4	Missing, low-confidence, incomplete-path, and restored evidence	The gate closes for incomplete or insufficient evidence; a later complete fresh basis reopens it	P4, P5
T5	Lower score with fresher sequence, including rollover	Fresher sequence wins	P5
T6	Equal sequence, different hop count	Lower-hop candidate wins	P5
T7	Provisional winner fails commit validation	Next feasible candidate is selected	P6
T8	Empty set and complete exhaustion	Exactly one native fallback is returned	P7
T9	Constant target under bounded update	Error decreases geometrically; state remains bounded	P1, P8
T10	Forward switch then smaller reverse change	Immediate reverse switch is rejected	P9

**Table 15:** Deterministic decision-semantic comparison of baseline AODV and the reference TA-BI adapter.

ID	Profile	AODV Outcome	TA-BI Outcome	Interpretation
E1	Equal sequence/hops; A lacks RouteBI; A has Trust 0.9; B is complete	A	A	Common full-basis gate closes; no asymmetric reward
E2	Equal sequence/hops; complete scores A 0.62, B 0.81	A	B	RouteScore changes only the native tie
E3	A has fresher sequence and lower score	A	A	Freshness remains authoritative
E4	A has fewer hops and lower score	A	A	Hop precedence remains authoritative
E5	TA-BI provisional winner fails commit-time validation	A	A	Next feasible candidate or native fallback is returned

**Table 16:** Four-level evidence taxonomy and inference boundary.

Evidence Class	Question Answered	Evidence in This Article	Explicit Non-Inference
Formal correctness	Does a proposition follow from declared assumptions and definitions?	P1–P9 and proofs	No topology-, radio-, workload-, or packet-level outcome
Executable conformance	Does deterministic code exercise the specified state, ordering, availability, and fallback branches?	S1–S3; T1–T10	No stochastic dynamics or performance superiority
Integration reachability	Were declared modules and route-lifecycle events observed to connect?	Historical S5 and S6 record	No independent rerun, protocol-equivalence proof, or quantitative performance inference
Empirical network performance	What end-to-end outcomes occur under repeated controlled scenarios?	Not evaluated	No PDR, delay, throughput, energy, overhead, scalability, or resilience claim

## Data Availability

Files S1–S3 are the rerunnable deterministic evidence set; S1 was validated with CPython 3.13.5, uses standard-library functionality only, and requires no random seed. File S4 supplies the literature coding sheet, and File S7 supplies execution instructions and SHA-256 checksums. Files S5 and S6 document a historical integration-reachability record. Its exact raw log, scalar/vector files, seed, and source snapshot are unavailable; it is therefore excluded from the rerunnable evidence set and supports no network-performance inference.

## Supplementary Materials

The following supporting information can be downloaded at: https://www.mdpi.com/article/10.3390/s26175422/s1. The labels S1–S7 correspond exactly to the distributed filenames: File S1, TA-BI_conformance_oracle.py; File S2, TA-BI_conformance_results.txt; File S3, TA-BI_decision_comparison.csv; File S4, TA-BI_literature_coding_sheet.csv; File S5, TA-BI_OMNeT_conformance_trace_summary.txt; File S6, SIMULATOR_ARTIFACT_AVAILABILITY.txt; and File S7, TA-BI_REPRODUCIBILITY_MANIFEST.txt.
